# A membrane-inserted structural model of the yeast mitofusin Fzo1

**DOI:** 10.1038/s41598-017-10687-2

**Published:** 2017-08-31

**Authors:** Dario De Vecchis, Laetitia Cavellini, Marc Baaden, Jérôme Hénin, Mickaël M. Cohen, Antoine Taly

**Affiliations:** 1 Institut de Biologie Physico-Chimique, Laboratoire de Biochimie Théorique, UPR 9080, Centre National de la Recherche Scientifique, Paris, France; 2 Institut de Biologie Physico-Chimique, Laboratoire de Biologie Cellulaire et Moléculaire des Eucaryotes, UMR 8226, Centre National de la Recherche Scientifique, Sorbonne Universités, UPMC University of Paris 06, Paris, France

## Abstract

Mitofusins are large transmembrane GTPases of the dynamin-related protein family, and are required for the tethering and fusion of mitochondrial outer membranes. Their full-length structures remain unknown, which is a limiting factor in the study of outer membrane fusion. We investigated the structure and dynamics of the yeast mitofusin Fzo1 through a hybrid computational and experimental approach, combining molecular modelling and all-atom molecular dynamics simulations in a lipid bilayer with site-directed mutagenesis and *in vivo* functional assays. The predicted architecture of Fzo1 improves upon the current domain annotation, with a precise description of the helical spans linked by flexible hinges, which are likely of functional significance. *In vivo* site-directed mutagenesis validates salient aspects of this model, notably, the long-distance contacts and residues participating in hinges. GDP is predicted to interact with Fzo1 through the G1 and G4 motifs of the GTPase domain. The model reveals structural determinants critical for protein function, including regions that may be involved in GTPase domain-dependent rearrangements.

## Introduction

The mitochondrial network is in constant motion, and its morphology is determined by the fusion and fission of mitochondrial membranes. This process, known as mitochondrial dynamics, is essential for the maintenance, function, distribution and inheritance of mitochondria, allowing the cell to respond to its ever-changing physiological conditions^1, 2^. Defects in mitochondrial dynamics are associated with neurological disorders and plasticity; thus, investigations into this subject are physiologically relevant^3^.

Mitochondria have evolved a set of large GTPases in the dynamin-related protein (DRP) family that constitute the fusion/fission apparatus. Among these DRPs, mitofusins (Mfn1 and Mfn2 in mammals) are dedicated to the homotypic tethering and fusion of mitochondrial outer membranes (OMs)^4, 5^. In addition, Mfn2 is also localized on endoplasmic reticulum (ER) membranes to regulate contacts between the ER and mitochondria^6–8^. Fzo1p (hereafter called Fzo1) is the sole mitofusin homologue in *Saccharomyces cerevisiae* ^9^. Fzo1 is embedded in the mitochondrial OM with a transmembrane (TM) region that spans the membrane twice, exposing the N- and C-terminal portions to the cytosol and a loop to the intermembrane space^10^. Fzo1 is characterized by an N-terminal GTPase domain flanked by two coiled-coil heptad repeats (HRs) (HRN and HR1, respectively)^9, 11^. The C-terminal region contains an additional HR (HR2).

Although the factors promoting the fusion of mitochondrial membranes were identified more than a decade ago, our understanding of the precise sequence of events that lead to the fusion of OMs has long been challenged by the lack of information regarding mitofusin structures. GTP hydrolysis was recently shown to be required to bring the OMs together prior to fusion^12^, and the integrity of the Fzo1 GTPase domain is essential for binding the Mdm30 ubiquitin ligase to the mitofusin^13^. These observations led to the hypothesis that GTPase domain-dependent rearrangements of Fzo1 presumably occur concomitantly with mitochondrial tethering^12–15^. These conformational changes would be comparable to the changes observed in the bacterial dynamin-like protein (BDLP), a protein related to mitofusins^16^. The BDLP structure exists in two conformational states: a “closed” compact structure observed upon GDP binding^16^ and an “opened” extended structure observed in the presence of a non-hydrolysable GTP analogue^17^. A similar two-state model may be hypothesized for mitofusins.

The structure of a human Mfn1 fragment (residues 1–364) containing the GTPase domain (residues 75–336) linked to the second half of the HR2 domain (residues 694–741) was recently solved and found to exhibit a fold that shares a striking similarity to the corresponding regions in BDLP^18, 19^. This long-awaited observation is not only consistent with the established homology between mitofusins and BDLP but also indicates that molecular modelling based on BDLP structures is an effective approach to investigate the architecture of full-length mitofusins^15, 20, 21^. However, previous models of mitofusins were not dynamically assessed in a membrane environment and lacked experimental validation.

Here, we present a full-atom homology model of Fzo1 in complex with GDP, which is based on the solved crystal structure of BDLP in the closed conformation^16^. As the proteins are distantly related, the modelling strategy integrated information from several template structures and published experimental data. The proposed Fzo1 model was simulated in three extended molecular dynamics (MD) simulations in a fully hydrated lipid membrane environment. Model consistency was validated by *in vivo* experiments using controlled site-directed mutagenesis targeting predicted interactions and comparison to partial crystal structures of human Mfn1.

The presented model provides structural insights at the residue level and will be instrumental in shedding light on the structural determinants of mitochondrial membrane fusion.

## Results and Discussion

### A near full-length and consistent mitofusin model based on BDLP

The template search identified BDLP from *Nostoc punctiforme* as the most suitable template for homology modelling of *S. cerevisiae* Fzo1 (structure 2J68, 3.1 Å^16^), as it shared 20% sequence identity and 43% similarity with Fzo1 (Supplementary Table 1). Several iterations were crucial for improving the stereochemical quality of the model, as shown by steady improvements in the stereochemical measures (Table 1).

**Table 1:** Stereochemical evaluation of the Fzo1 model during the modelling procedure.

	First alignment	Manually refined alignment	Loop modelling and structural refinement
% Most favoured	89/83.9	93/90.9	93.8/90.9
% Additional allowed	7.9/14.9	4.3/7.8	6.2/8.9
% Outliers	3.1/1.3	2.7/1.3	0/0.2
RMSD (Å)	0.9	0.7	0.6

Percentage of residues within the different Ramachandran plot regions are indicated. The values were determined using MolProbity^22^/ProCheck^23^. The RMSD was computed using UCSF Chimera^24^ after the superposition of the BDLP crystal structure and Fzo1 model for each of the iterations indicated.

Although homologous regions were detected throughout the amino acid sequences of the target and template, the N-terminal region showed greater differences between the two species. This divergence is not surprising because, unlike other mitofusin family members, Fzo1 possesses a unique heptad repeat (HRN) upstream of the GTPase domain^2^. Furthermore, our analysis using different alignment algorithms showed that the Fzo1 sequence aligns well to the template starting at approximately residue 100 (Supplementary Figs 1–4). Secondary structure predictors (Supplementary Fig. 5) described the region upstream of HRN as unstructured, and the first 86 N-terminal residues were predicted to be disordered by the DISOPRED3 algorithm^25^ (Supplementary Fig. 6).

**Figure 1:**
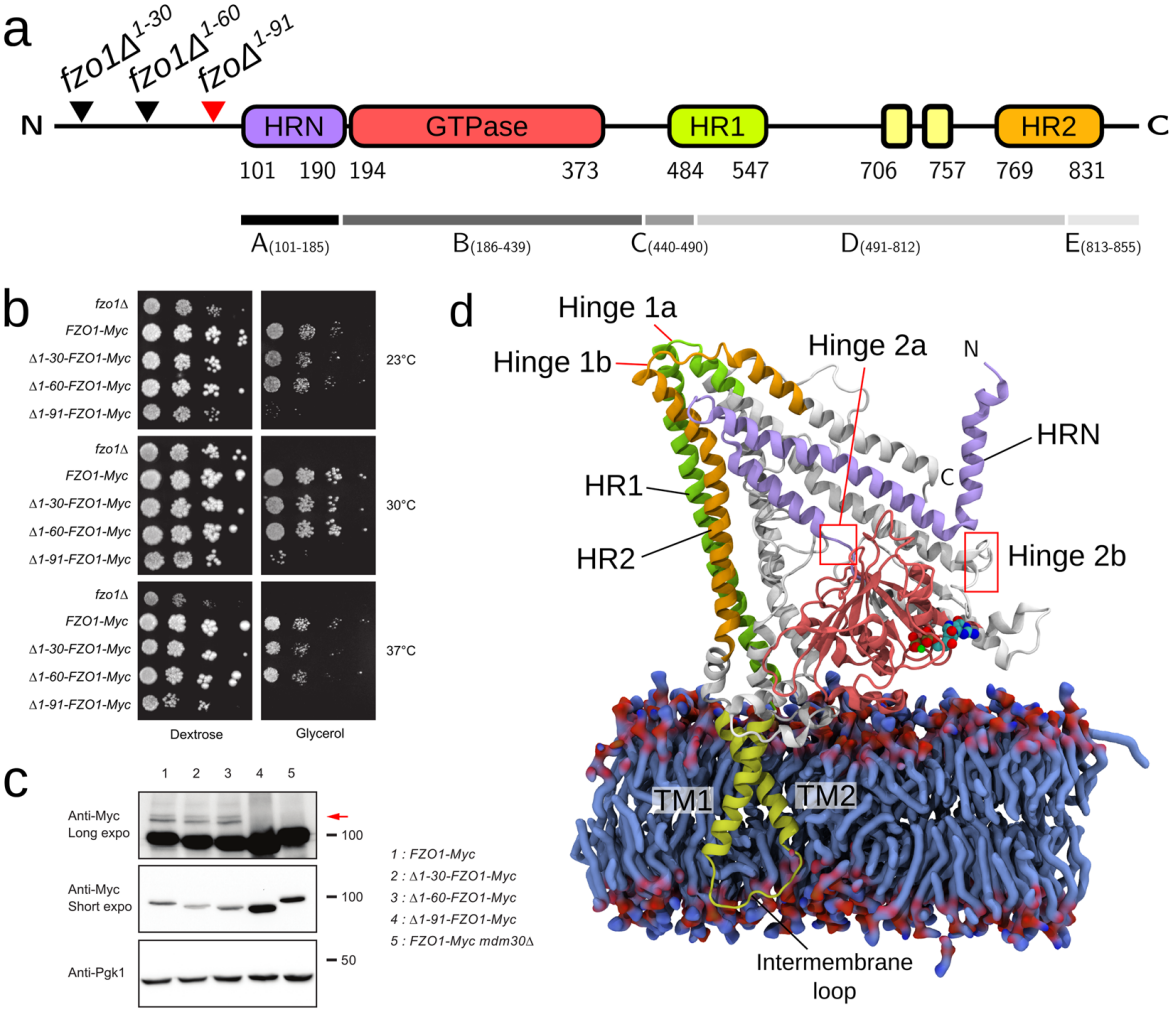
Architecture and domain organization of the Fzo1 model. (**a**) Scheme showing the domains of Fzo1 from *S. cerevisiae*. Residue numbers for each domain and the deletion mutants are indicated. The *red* arrow highlights the deletion mutant that causes a defect in respiration. HRN, N-terminal truncated heptad repeat (*violet*, residues 101–190); GTPase, GTPase domain (*red*, residues 194–373); HR1, heptad repeat 1 (*green*, residues 484–547); transmembrane segments (*yellow*, residues 706–757), HR2, heptad repeat 2 (*orange*, residues 769–831). The fragments between the hinges are indicated below the alignment and are designated A to E. (**b**) Dextrose and glycerol growth spot assay for the 30-, 60- and 91-amino acid N-terminal *FZO1* deletion mutant strains, namely *fzo1Δ* ^*1–30*^, *fzo1Δ* ^*1–60*^ and *fzo1Δ* ^*1–91*^, respectively. The *fzo1Δ* and *FZO1-Myc* strains were used as negative and positive controls, respectively. (**c**) Anti-Myc and Anti-Pgk1 immunoblots of whole-cell extracts prepared from the strains used in (**b**). The *FZO1-Myc mdm30Δ* strain was used as a control for lack of Fzo1 ubiquitylation. Molecular weight markers are indicated on the right of long or short exposures of the immunoblots. (**d**) Fzo1 model after the equilibration phase (as described in the Methods section). The *blue* surface represents POPE and POPC lipids from a portion of the bilayer. The GDP nucleotide and the Mg^2+^ ion are depicted in the space-filled representation.

We took advantage of the requirement for Fzo1 during respiration^9, 26^ to obtain insights into the role of the first 86 N-terminal residues upstream of the Fzo1 HRN domain. Thus, we assessed the fermentative (glucose) and respiratory (glycerol) growth capacities of three distinct N-terminal deletion mutant strains of *FZO1*, namely *fzo1Δ* ^*1–30*^, *fzo1Δ* ^*1–60*^ and *fzo1Δ* ^*1–91*^ as compared to positive (wild-type) and negative (*fzo1Δ*) control cells (Fig. 1b). Although the *fzo1Δ* ^*1–30*^ and *fzo1Δ* ^*1–60*^ mutants displayed normal growth on both glucose and glycerol-containing media, the respiratory growth of the *fzo1Δ* ^*1–91*^ mutant was nearly completely abolished (Fig. 1b). Interestingly, the steady-state level of the *fzo1Δ* ^*1–91*^ protein was significantly increased compared with that in the wild-type *Fzo1* or the *Fzo1Δ* ^*1–30*^ and *Fzo1Δ* ^*1–60*^ mutants (Fig. 1c), suggesting that the deletion of residues 61 to 91 induces stabilization of the mitofusin, possibly by inhibiting Mdm30-mediated degradation^13^. Consistent with this observation, Mdm30-mediated ubiquitylation of *Fzo1Δ* ^*1–91*^ was undetectable (Fig. 1c). These results indicate that residues 61 to 91 of Fzo1 are essential for Fzo1 function and its ubiquitin-dependent regulation by Mdm30. These phenotypes mimic the previously observed phenotypes following inactivation of the GTPase domain^13^, suggesting that residues upstream of the HRN may participate in regulating GTP binding or hydrolysis by the mitofusin.

Despite the previously unappreciated functional importance of residues 61 to 91, the lack of a template for the region upstream of the HRN domain and the resulting uncertainty in its structure assignment imposed omitting the first 100 amino acids of Fzo1 from the final model. At this stage, we are tempted to speculate that together with the N-terminal helix observed in the model presented below (Helix α1 in the HRN), residues 61 to 100 may fall back on the GTPase domain to regulate its activity, although further investigation is required.

The Fzo1 model and its essential domains are illustrated in Fig. 1d. Most of the protein is exposed to the cytoplasmic side of the lipid bilayer and presents a similar architecture to BDLP, in which the stalk domain is composed of two four-helix bundles connected by hinges^16^ (Fig. 1d). This organization is consistent with the recently solved minimal GTPase domain structures from human mitofusin Mfn1, in which the N- and C-terminal region of the GTPase domain form a four-helix bundle together with the HR2 helix that follows hinge 1b^18, 19^. Since our Fzo1 model was built before the partial Mfn1 structures were published, this similarity validates the homologous regions in our model and provides substantial confidence in domains of the molecule that are not present in the Mfn1 fragment (*e.g*., the TM region, the trunk and hinges 1a/1b).

### The Fzo1 model has a stable core when simulated in a membrane environment

The conformational stability of the Fzo1 model was assessed in three 500-ns MD trajectories in a mixed lipid bilayer environment (referred to as Fzo1.I, Fzo1.II and Fzo1.III).

**Figure 2:**
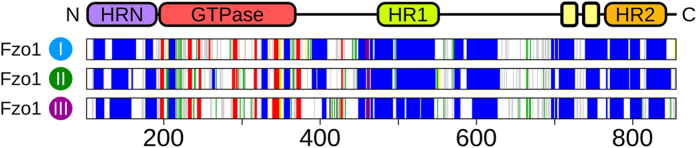
Stable secondary structure motifs in simulations. Comparison of the Fzo1 domain organization shown in Fig. 1a with the stable secondary structures (persistence greater than 90%) in the three replicate simulations. The colour code is *blue*, α-helix; *red*, β-sheet; *yellow*, turn; *green*, bend; *black*, β-bridge; and *violet*, π-helix.

The predominant secondary structure of the Fzo1 model confirms a coherent and stable architecture between the three simulations (Fig. 2), with a clear pattern of long α-helices retained during the time course of the simulation, notably for the predicted coiled-coil domains HRN, HR1 and HR2, as well as for the TM dimer.

Supplementary Fig. 7 and Supplementary Table 2 show the drift of the protein structure from the energy-minimized initial model as time series of the RMSD. The high RMSD values observed for the whole protein in the last frame (5.7–7.3 Å) account for significant local structural deviations (Supplementary Fig. 7, top panels, *black* lines). The decomposition into protein segments (Supplementary Fig. 7, top panels) reveals that these high values are mostly due to i) unstructured regions, ii) portions of the structure that possess high flexibility, such as the N-terminal helices, as measured by the RMSF (Supplementary Fig. 7, top panels, *red* lines), and iii) regions that are unresolved in the template crystal structure, *i.e*., residues 502–510 (residues 631–653 in the Fzo1 model). When the contribution of these components is removed, a lower RMSD with a plateau between 4–5 Å is observed (Supplementary Fig. 7, top panels). The analysis of the *ab initio* modelled TM domain shows the same average RMSD value of 2.4 Å (Supplementary Table 3), indicating stability and consistency among the three trajectories.

Together, these results indicate consistency among the trajectories that show a similar fluctuation profile (Supplementary Fig. 6) with the model that underwent the equilibration and production stages, maintaining the proposed fold.

#### Fzo1 transmembrane domain

The highest-scoring model constructed using PREDDIMER^27^ shows a compact structure characterized by a crossing angle (χ) of 119.7° (other models are shown in Supplementary Fig. 8). The second TM helix (TM2) harbours a GxxxG motif (with x representing any amino acid) that was previously reported to play a role in helix dimerization^28, 29^. We based our choice on the observation that interactions with (small)xxx(small) motifs are often associated with the formation of right-handed dimers characterized by negative helix-helix crossing-angles in parallel dimers^30–32^. Here we have a right-handed antiparallel dimer, with χ = 120° (Supplementary Fig. 8). This is currently the most plausible arrangement. The second-highest scoring model has a similar score and involves the same dimerization motif, although with a straighter geometry (χ = 175°, nearly antiparallel). While the latter arrangement is less common it cannot be excluded and the reader should be aware that alternative transmembrane arrangements are possible.

According to the RMSD analysis for each MD simulation trajectory (Supplementary Fig. 7), the TM domain is well anchored and does not undergo notable structural changes, as confirmed by the analysis of the dominant secondary structure (Fig. 2). The observed fluctuation values are similar in the three replicates (Supplementary Fig. 6). These features suggest that the structure of the TM domain is realistic.

To characterize the interaction between the two TMDs in more details than just pure geometrical contacts, we analysed specific hydrogens bonds as described in methods. All simulations indicated a hydrogen bond between the two TM helices that involved the side-chains of Lys716 and Ser746 (38%, 19% and 18% persistence for Fzo1.I, Fzo1.II and Fzo1.III, respectively). Notably, Ser746 resides on the TM2 helix and belongs to the aforementioned GxxxG motif.

**Figure 3:**
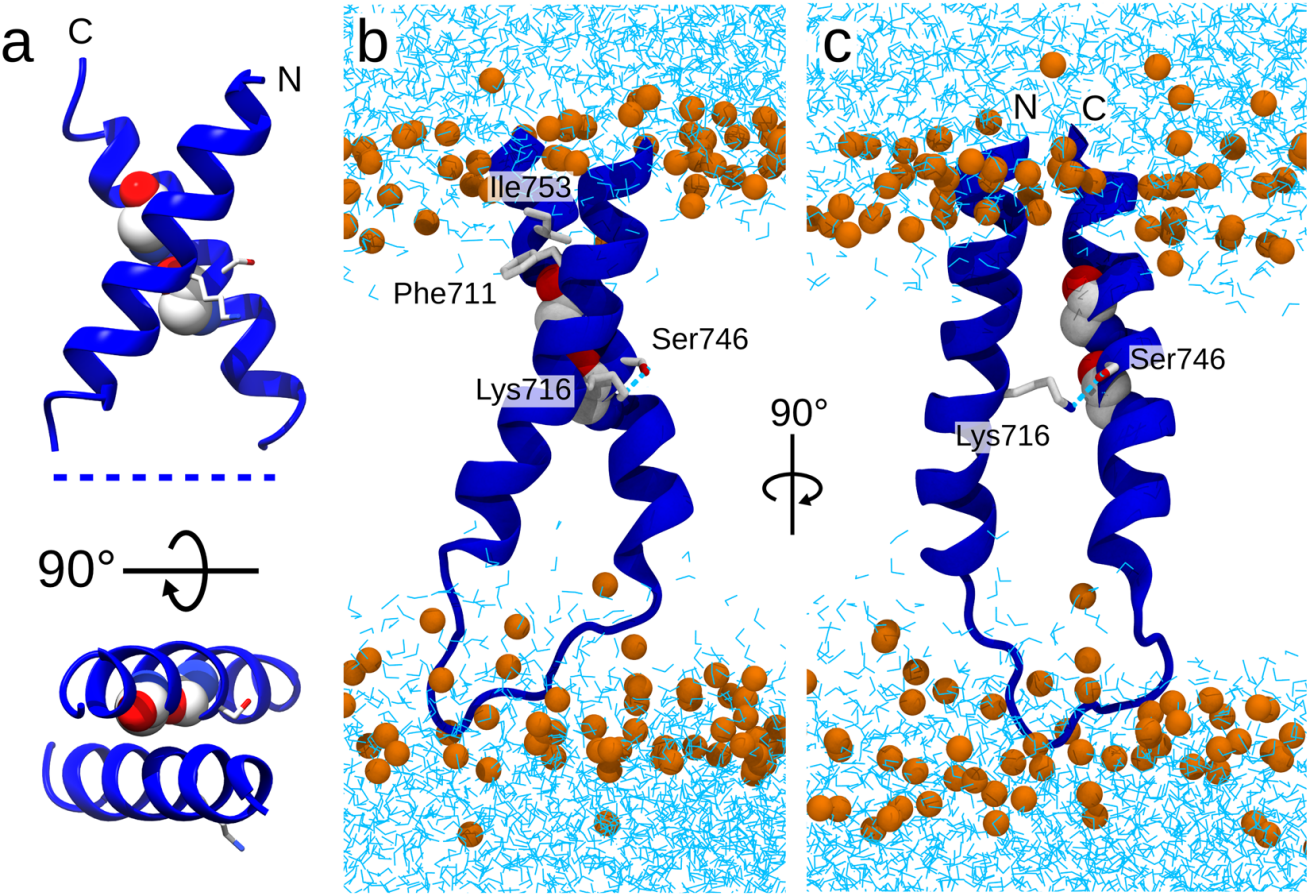
Insights into the Fzo1 transmembrane domain. (**a**) PREDDIMER^27^ *ab initio* prediction of the helical dimer (*F*_*SCOR*_ 3.113, crossing angle χ 119.7°). (**b**,**c**) Snapshots from the Fzo1.I trajectory showing the most representative structure (*i.e*., the centroid). Glycine residues within the GxxxG motif are presented as a space-filled representation, whereas residues involved in interactions are depicted in stick form. Phosphate atoms from POPC and POPE (*orange*) and water molecules (*cyan*) are indicated, whereas the rest of the protein is omitted for clarity.

Analysis of the contact residues in the TM dimer revealed an abundance of promiscuous stabilizing interactions (Supplementary Table 4). The only unambiguous interaction seemed to be Phe711-Ile753, whereas the remainder of the interactions varied among the trajectories. After manually adjusting the side-chain rotamer (see Methods), the Ile753 residue on helix TM1 directly faced residue Phe711 on TM2, increasing the packing of the TM helices (Fig. 3b). The analysis of protein-lipid hydrogen-bonding indicated that Fzo1 had more interactions with POPE compared to POPC (Supplementary Table 5). Given that the bilayer contains both lipids in the same proportion, this observation suggests a preference for POPE.

### Experimental validation of the Fzo1 model

The yeast mitofusin Fzo1 has previously been characterized by introducing many mutations across its functional domains (Supplementary Fig. 9). We aimed to evaluate the relationship between our structural model and the wealth of published data available, as well as the additional targeted mutations summarized in Supplementary Table 6 and discussed hereafter. This analysis was performed on the most representative structure (i.e., a centroid) identified after a cluster analysis of the Fzo1.I trajectory (see the Methods).

**Figure 4:**
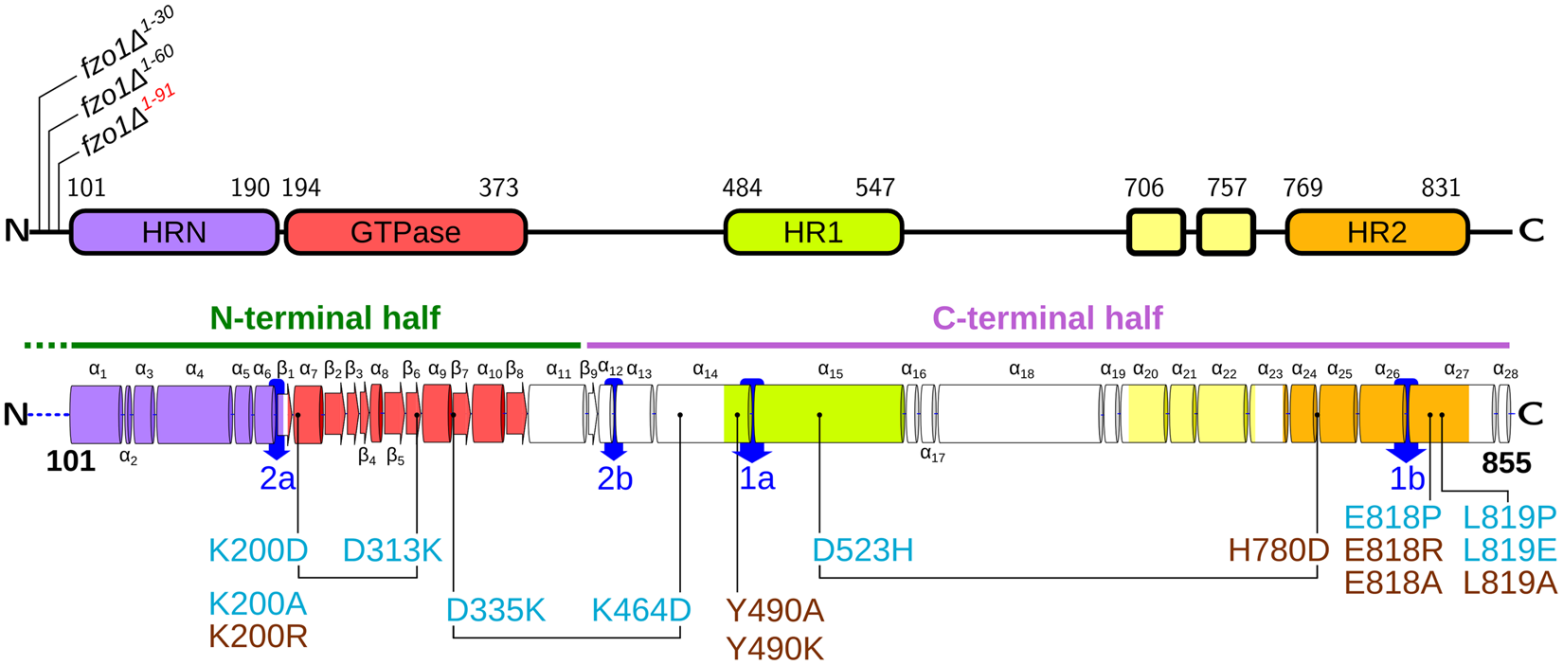
Cartoon representation of the Fzo1 model and its functional domains. (top) Residue numbers delimiting the domains, (bottom) secondary structure elements are annotated with the Fzo1 mutations performed in this study. Mutants considered for the charge swap strategy are connected by a bar. The colour code is *cyan*, loss of function (LOF) and *maroon*, wild-type phenotypes. Putative hinge regions are indicated by *blue* arrows. Previously reported mutations across Fzo1 functional domains are shown in Supplementary Fig. 9. *Green* and *pink* horizontal bars above the secondary structure elements depict the N- and C-terminal halves, respectively. The topology diagram was generated using the HERA tool^33^.

Figure 4 shows the secondary structure elements. The organization of the HR domains in our model reveals a more complex organization than initially hypothesized, as these repeats are not merely assembled as continuous helices. In particular, HRN, HR1 and HR2 are characterized by a complex structure and are composed of several tandem helices. This observation may be key to interpreting the functional outcomes of several Fzo1 mutations. For instance, according to our model, previously uncharacterized regions, such as the α14 and α27 helices, may contribute to protein function. Although these helices are not defined by any heptad periodicity, they represent a structural continuation of the HR1 and HR2 repeats, separated by the hinges 1a and 1b, respectively (Fig. 4, blue arrows). Consequently, helices α14 and α27 are located parallel to the HRN in our 3D model (Fig. 1d). This configuration is strikingly similar to the four-helix bundle observed in the recent crystal structure of an Mfn1 fragment (Supplementary Fig. 10), in which the C-terminal helix has been proposed to stabilize the identified bundle^18^. Here, we propose that Fzo1 helix α27 (Fig. 4) plays a similar role (Supplementary Fig. 10). Consistent with this observation, deletion of the last 24 residues of Fzo1 (Fzo1 ∆826–855) was previously shown to abolish mitochondrial fusion^34^. Although the reconstitution of helix α27 in our model (res 815–845) results from the manual modification of the target-template alignment (see Methods), the considerations described above provide a significant validation of our modelling approach.

Based on these observations, we refined our analysis of the model using a series of site-directed mutagenesis experiments across the predicted interactions revealed during the MD analysis.

#### Interactions between the N- and C-terminal halves of the protein

**Figure 5:**
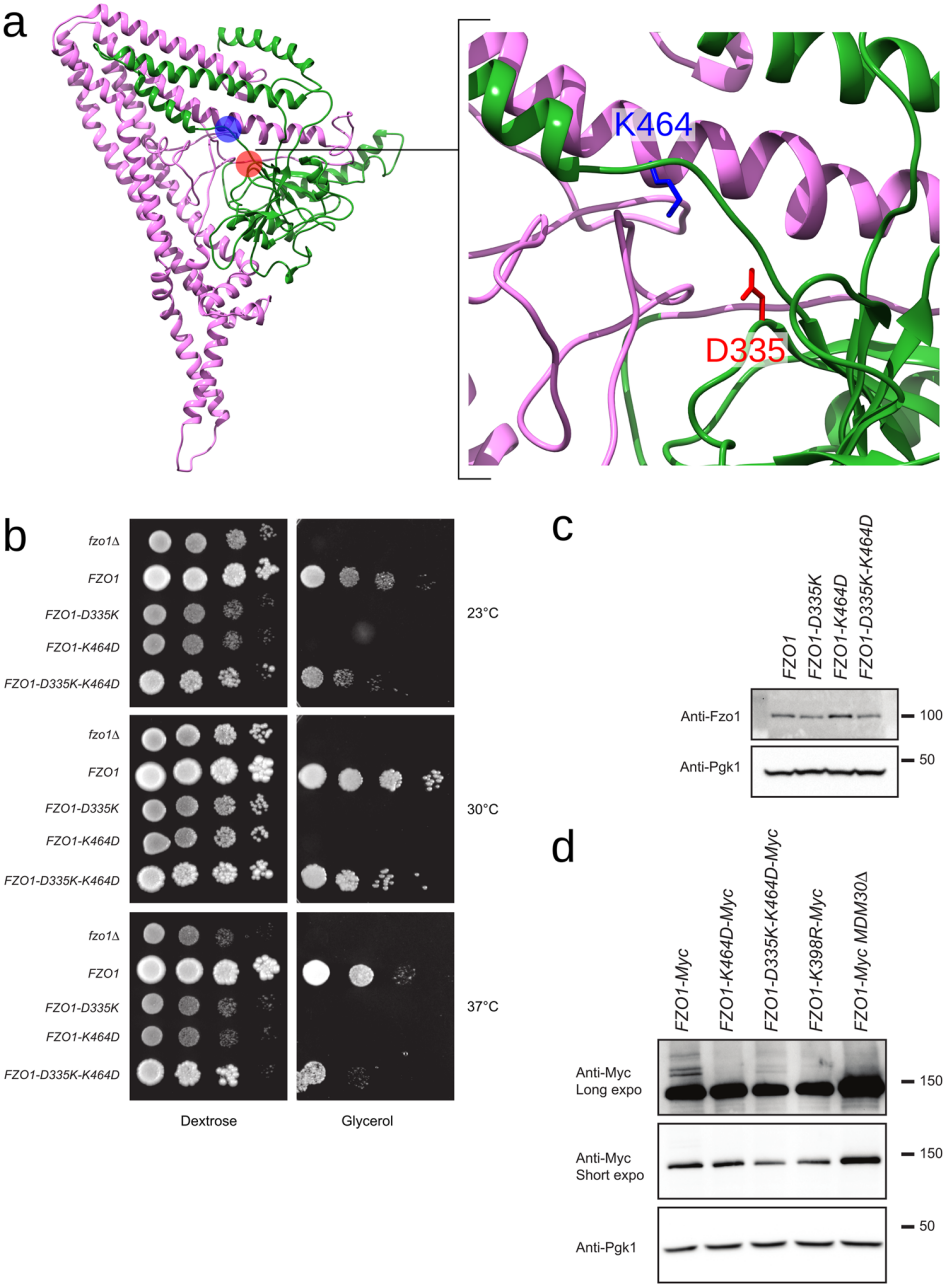
Swap mutations across the predicted salt bridge D335-K464. (**a**) Relative positions of D335 (*red*) and K464 (*blue*) in the Fzo1 model. The *green* and *pink* regions correspond to the N- and C-terminal halves, respectively (Supplementary Fig. 9). (**b**) Dextrose and glycerol growth spot assay. (**c**) Anti-Fzo1 and anti-Pgk1 immunoblots of whole-cell extracts prepared from the strains analysed in (**b**). Molecular weight markers are indicated on the right. (**d**) Anti-Myc and Anti-Pgk1 immunoblots of whole-cell extracts prepared from the indicated strains. Molecular weight markers are indicated on the right of long or short exposures of the immunoblots.

We searched for salt bridges to test experimentally the putative interactions and validate the proposed model for Fzo1. We focused our attention on the predicted salt bridge D335-K464 (Fig. 5) because the interaction was identified in the model after the minimization phase and is conserved in BDLP, where both charged residues are inverted (His202 and Glu331, respectively, Supplementary Fig. 11). We maintained the focus on D335-K464, even though the interaction was weakened in subsequent simulations (1.5%, 2% and 27% for Fzo1.I, Fzo1.II and Fzo1.III, respectively). This choice was motivated by the following observations: a preliminary MD study suggested that the salt bridge was present in 61% of structures, and the solved fragment from Mfn1 shows an interaction between the homologous pair of charged residues, Asp200 and Lys336^18, 19^ (Supplementary Fig. 10). In addition, these mutants provide us with the opportunity to investigate the importance of the Lys464 residue, which is a target for post-translational modification by ubiquitin in Fzo1^15^.

We employed a charge swap strategy to assess this predicted interaction, which relies on the assumption that a double charge reversal mutation across a predicted salt bridge should restore a wild-type phenotype, whereas single point mutations may abolish the salt bridge, therefore affecting protein function. Although the single point mutations *D335K* and *K464D* induced a total inhibition of respiratory growth, this strong defect was partially but significantly corrected by the swap mutation *D335K-K464D* (Fig. 5b), thus confirming the proximity between residues at position 335 and 464. Consistent with the role of K464 in the ubiquitin-mediated degradation of Fzo1^15^, the level of the *Fzo1 K464D* mutant protein increased compared with that of the wild-type control. Both the *Fzo1 D335K* single mutant and, most surprisingly, the *D335K-K464D* double mutant (charge swap) displayed levels comparable to the wild-type protein. This observation led to compare the ubiquitylation status of single (*K464D*) or double swap (*D335K-K464D*) Fzo1-13Myc mutants with that of *K398R* that is established to alter ubiquitylation of the mitofusin^15, 35^. As expected, detection of the Mdm30-dependent ubiquitylation doublet was strongly impaired in *K464D* and *K398R* mutants (Fig. 5d). Strikingly, however, the double swap mutation *D335K-K464D* restored partial ubiquitylation of Fzo1-13Myc (Fig. 5d). As aspartate cannot be ubiquitylated, the swap mutations may thus also allow position 335 to be ubiquitylated by restoring the proximity of residues 335 and 464. Alternatively, this restored proximity may contribute to maintain a correct fold of Fzo1 that would favor ubiquitylation on K398. The latter possibility is more likely as K398 is the main target for Mdm30-dependent ubiquitylation of Fzo1^35^. In aggregate, these results provide experimental confirmation of a physical interaction between the two residues located in the N- and C-terminal halves of Fzo1 and contribute to validating the predicted proximity in our model.

In order to further cross-validate our model, we have constructed a new partial Fzo1 model based on human Mfn1 crystal^19^ (Supplementary Fig. 10). We compared key interactions predicted by our BDLP-based model with the alternative partial model. Interestingly, among the numerous interactions conserved (Supplementary Fig. 10), the predicted interaction between K464 and D355 (Fig. 5), is also observed in the Mfn1-based model.

#### Electrostatic interactions are involved in organizing the HR domain

The HR regions in Fzo1 are required for mitofusin architecture, showing LOF phenotypes upon disruption of their presumed helical folds imposed by proline mutations^11^. In our model, the coiled-coil HR1 and HR2 domains form an antiparallel bundle (Fig. 1d). Yet surprisingly, we did not observe the typical HR interaction pattern in the HR1/HR2 coiled-coil structure (Supplementary Table 7 and Supplementary Fig. 12). The hydrophobic spine of HR2 is solvent-exposed, and the HR1 hydrophobic spine actively interacts with the nearby α18 helix (residues 584–630, Fig. 4 and Supplementary Fig. 12), which interestingly does not exhibit heptad periodicity. Consequently, the HR2 repeat may be available for putative interactions between Fzo1 molecules similarly to human Mfn1, in which the HR2 domain dimerize through anti-parallel binding^36^. Whether these interactions take place in *trans* (between Fzo1 molecules from opposing membranes) as previously proposed^20, 36^ or in *cis* (between Fzo1 molecules from the same membranes) prior to mitochondrial tethering^37^ is an important question that will need future clarification.

**Figure 6:**
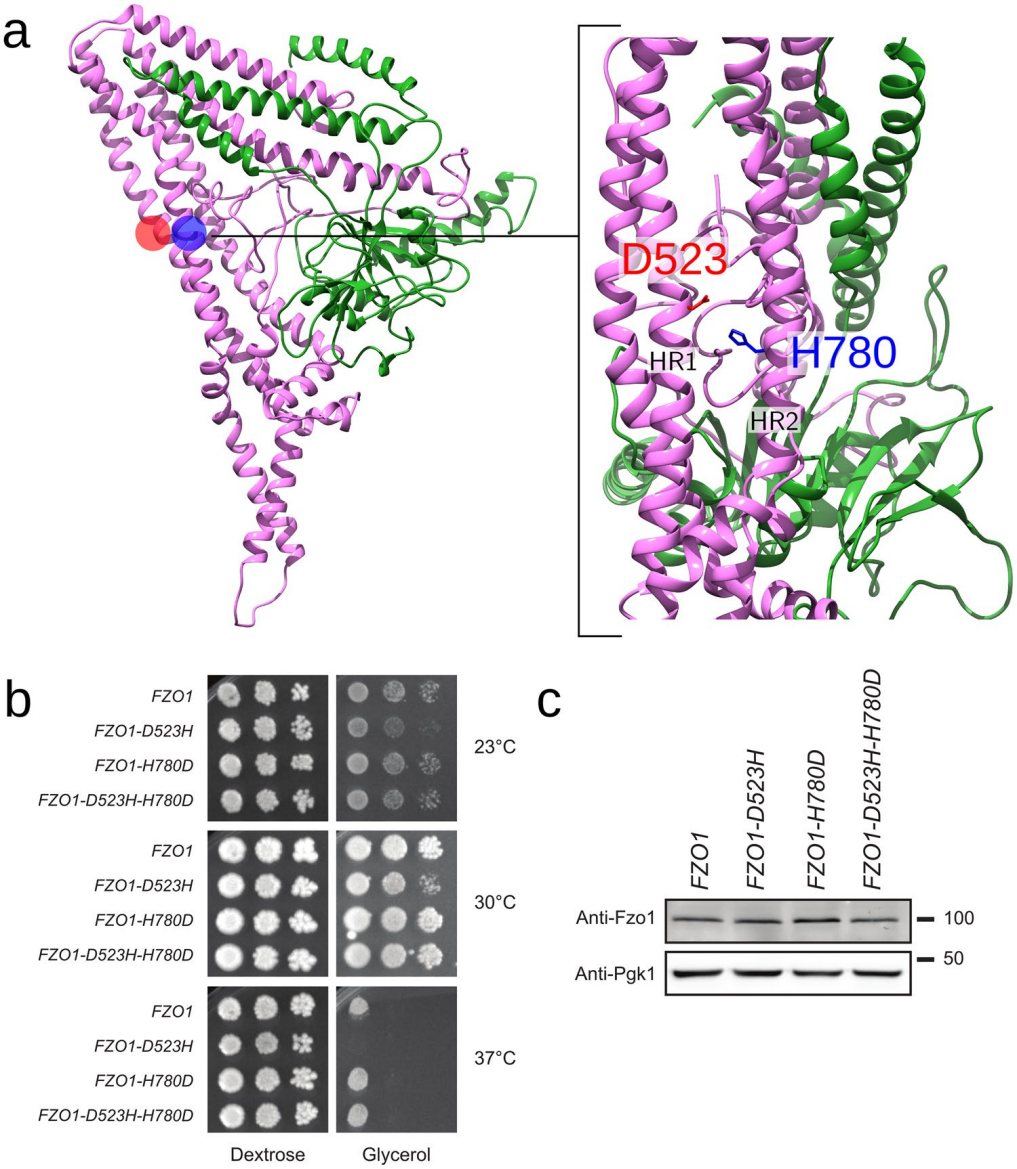
Swap mutations across the predicted salt bridge D523-H780. (**a**) Location of D523 (*red*) and H780 (*blue*) in the Fzo1 model. The *green* and *pink* regions correspond to the N- and C-terminal halves, respectively (Supplementary Fig. 9). The positions of HR1 and HR2 are indicated. (**b**) Dextrose and glycerol growth spot assay. (**c**) Immunoblots of whole-cell extracts prepared from the strains analysed in (**b**). The Pgk1 immunoblot was used as a loading control. Molecular weight markers are indicated on the right.

In contrast to the lack of a classical pattern of hydrophobic interactions, we observed a network of intra- and interhelical salt bridges in the simulations. According the convention of labelling residues in a heptad repeat from *a* to *g*, with *a* and *d* usually indicating hydrophobic residues^38^, intrahelical interactions were observed in all three domains, with the majority of the *b-e* type, similar to the pattern previously observed for the neuronal SNARE complex^39^. Almost all interhelical salt bridges connected HR1 and HR2, with one exception connecting HRN and HR2 (observed only in Fzo1.II). The 8 salt bridges that persisted in all three trajectories are listed in Supplementary Table 8. We used site-directed mutagenesis to investigate a possible interhelical salt bridge between the HR1 and HR2 domains, the D523-H780 salt bridge, which was identified early on as having high persistence in a preliminary simulation. Indeed, although the distance rapidly rises above the adopted interaction cut-off of 6.5 Angstrom, the distance between its charged groups remains constant below 10 Å during the simulation. The charge swap strategy applied to these two residues revealed that the *D523H* point mutation caused a temperature-dependent respiratory growth defect. However, the H780D mutant yields a wild-type phenotype (Fig. 6b). Although this result may seem difficult to rationalize, an interpretation based on common observations with charge reversal mutations is that D523 and/or D780 may interact with more than one partner, allowing for a form of compensation when H780 is mutated. Strikingly, the swap mutant *D523H-H780D* induced complete rescue of the respiration defect caused by the *D523H* mutation. This result is consistent with the proximity between HR1 and HR2 in the Fzo1 model and suggests that this proximity is a requirement for mitofusin activity. Although the predicted salt bridge D523-H780 (HR1 and HR2, respectively) shown in Fig. 6 is not directly conserved, it is located near a salt bridge identified in the template structure of BDLP (D523 and H780 in Fzo1 correspond to I390 and F624 in BDLP, next to bridging residues R393 and D625; see the sequence alignment in Supplementary Fig. 11).

A recent study hypothesized that the HR2 domain may dissociate from the HR1 domain in active forms of Mfn2 and unfold itself from the rest of the protein to become available for *trans* interactions with exposed HR2 domains of Mfn2 molecules from opposing mitochondria^20^. However, a dissociation of one of the repeats seems unlikely because our model features tight interactions between the coiled-coil HR1 and HR2 domains, which appear to be required for maintaining the mitofusin fold (Fig. 1d).

#### Functional importance of the regions surrounding hinges 1a and 1b in the Fzo1 model

A tempting alternative hypothesis to the mitofusin conformational switch mediated by HR2 extension is the possibility that mitofusins perform extensive GTPase domain-dependent rearrangements through hinges 1a and 1b (Fig. 4), similar to BDLP^17^. In this regard, Mdm30 was previously shown to bind the N-terminal half of Fzo1 (HRN/GTPase domain) in a manner that required the GTPase domain-dependent displacement of the C-terminal half (HR1/TM/HR2 domain)^13^. Mutations within the HR2 domain that disrupt the interaction between the N- and C-terminal halves of Fzo1^11^ were thus expected to promote constitutive Mdm30 binding. Consistent with this hypothesis, the *L819P* mutation induced an increase in Mdm30-dependent ubiquitylation of Fzo1 and markedly accelerated the degradation of the mitofusin^13^.

**Figure 7:**
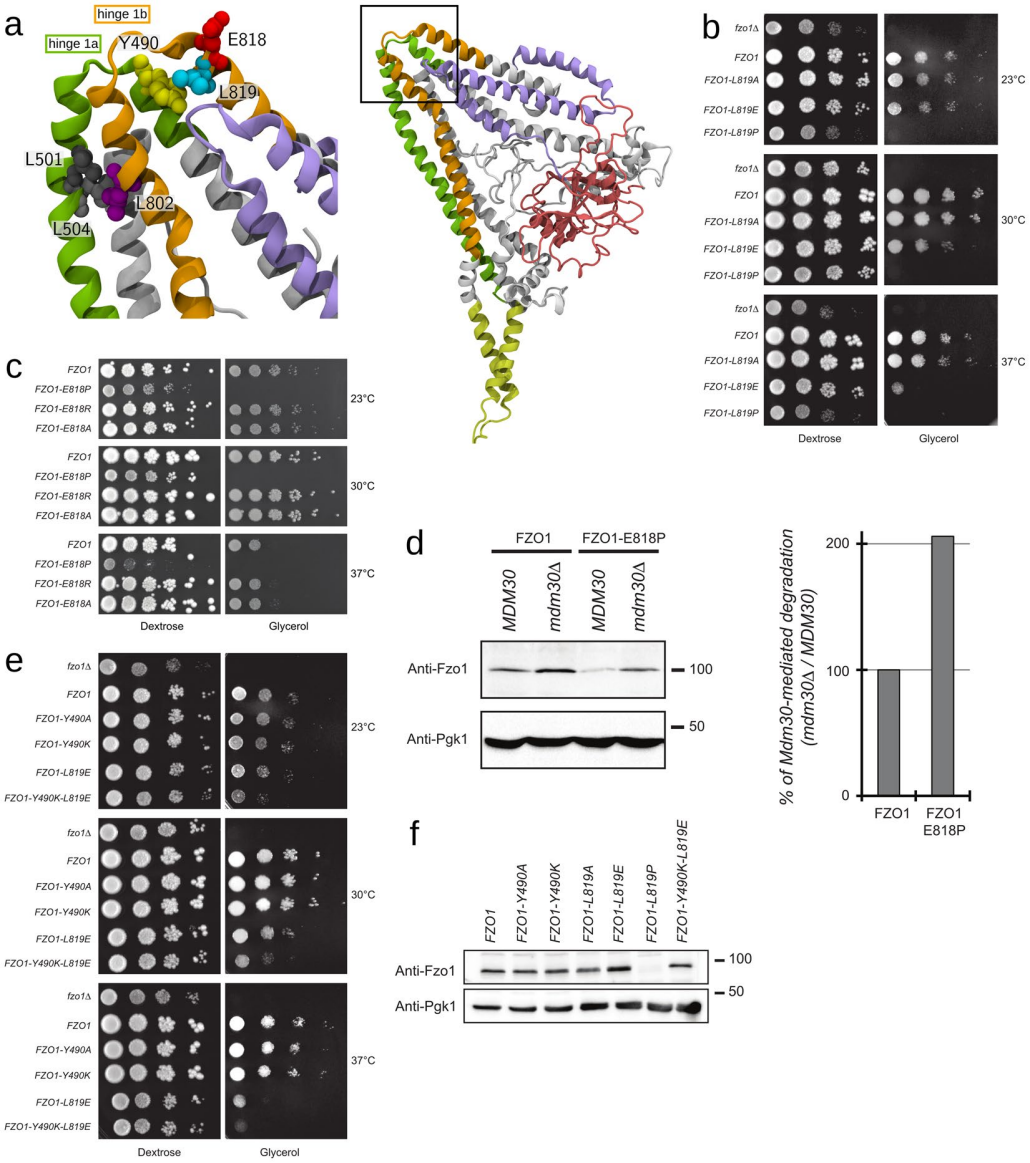
Critical residues in the Fzo1 model hinge region. (**a**) Detailed structure of the putative hinge region. The colour code is: *cyan*: Leu819, *yellow*: Tyr490, *purple*: Leu802, *red*: Glu818, *grey*: Leu501, and *light grey*: Leu504. The domains are: *violet:* HRN, *green:* HR1, and *orange*: HR2. The location of this hinge region within the model is presented in the right panel. **(b)**, **(c)** and **(e)** Dextrose and glycerol growth spot assay with indicated strains. **(d)** and **(f)** Anti-Fzo1 and anti-Pgk1 immunoblots of whole-cell extracts prepared from the strains used in (**b**), (**c**) and (**e**). Molecular weight markers are indicated on the right of immunoblots. The graph in (**d**) represents the quantified ratio of Mdm30-dependent degradation for wild-type Fzo1 or the Fzo1 *E818P* mutant.

The Leu819 residue is located on helix α27 of the Fzo1 model, with its side chain facing the helix α14 (Fig. 7a). Consistent with this arrangement, introduction of a negative charge at position 819 (*L819E*) impaired respiratory growth at 30 and 37 °C whereas replacement with an uncharged residue (*L819A*) did not have any effect (Fig. 7b). As previously shown, the *L819P* mutation abolished respiration at all temperatures (Fig. 7b) and enhanced degradation of Fzo1 (Fig. 7f). The location of Leu819 in hinge 1b suggests that the *L819P* mutation may induce a kink in this region that would in turn favor a conformational rearrangement of the mitofusin. To explore this possibility, we reasoned that perturbing the backbone conformation around Leu819 should also promote conformational remodelling of Fzo1 and mimic the *L819P* mutation effect. In this regard, Glu818 is predicted to lie in hinge 1b, with a water-exposed side-chain. Removing the side-chain charge (*E818A*) or even reversing it (*E818R*) did not affect respiratory growth, indicating that this position is not sensitive to modifications of the side-chain. However, we confirmed that this position is sensitive to backbone effects by introducing a proline residue (*E818P*) that completely abolished yeast growth on glycerol media (Fig. 7c). Moreover, while the E818P mutation induced an Fzo1 decrease that does not depend on Mdm30 (Fig. 7d; compare lanes 2 and 4), this mutant was also degraded by the Mdm30-mediated pathway twice more than wild-type Fzo1 (graph Fig. 7d). This observation is consistent with the effects of *L819P* and the potential conformational switch it exerts on Fzo1.

Tyr490 is located in hinge 1a in our model and is predicted to interact with Leu819 in Fzo1.I and Fzo1.II (75% and 61%, respectively) (Fig. 7a). Similar to *L819A* but in contrast to *L819E*, mutation of Tyr490 in Alanine (*Y490A*) or Lysine (*Y490K*) did not affect respiratory growth (Fig. 7e). However, combining *Y490K* with *L819E* (*Y490K-L819E*) induced an enhanced defect in respiration as compared to *L819E* (Fig. 7e) without affecting Fzo1 levels (Fig. 7f). This confirms the predicted proximity between Tyr490 and Leu819. Moreover, the *Y490P* mutation was previously reported to cause total inhibition of respiratory growth and induce the formation of loose mitochondrial aggregates that were hypothesized to result from a tethered trapping state^11^. Similar to hinge 1b, a mutation of Tyr490 to proline within hinge 1a may thus affect mitochondrial fusion by inducing conformational rearrangements in Fzo1.

Single point mutations, L501A and L504A, of residues located on the other side of the hinge were previously shown to be functionally silent but abolished mitochondrial fusion in combination^11^. Thus, these mutations were hypothesized to affect hydrophobic interactions, as both leucines were predicted to occupy the *a* and *d* positions in the HR1 domain^11^. The analysis of the contacts within our model revealed that these two leucine residues may in fact belong to a leucine cluster between residues Leu501, Leu504 and Leu802, which is located in close vicinity to hinge 1b (Fig. 7a). This cluster may explain the differential phenotypes obtained with the L501A or L504A mutants, as Leu802 may compensate for the single mutation but not the double point mutations.

Thus, these results highlight the critical importance of regions surrounding hinges 1a and 1b in Fzo1 function and suggest that they may be involved in mitofusin rearrangements.

### Atomistic insights into the GTPase domain

#### Binding site architecture and protein-GDP interactions

**Figure 8:**
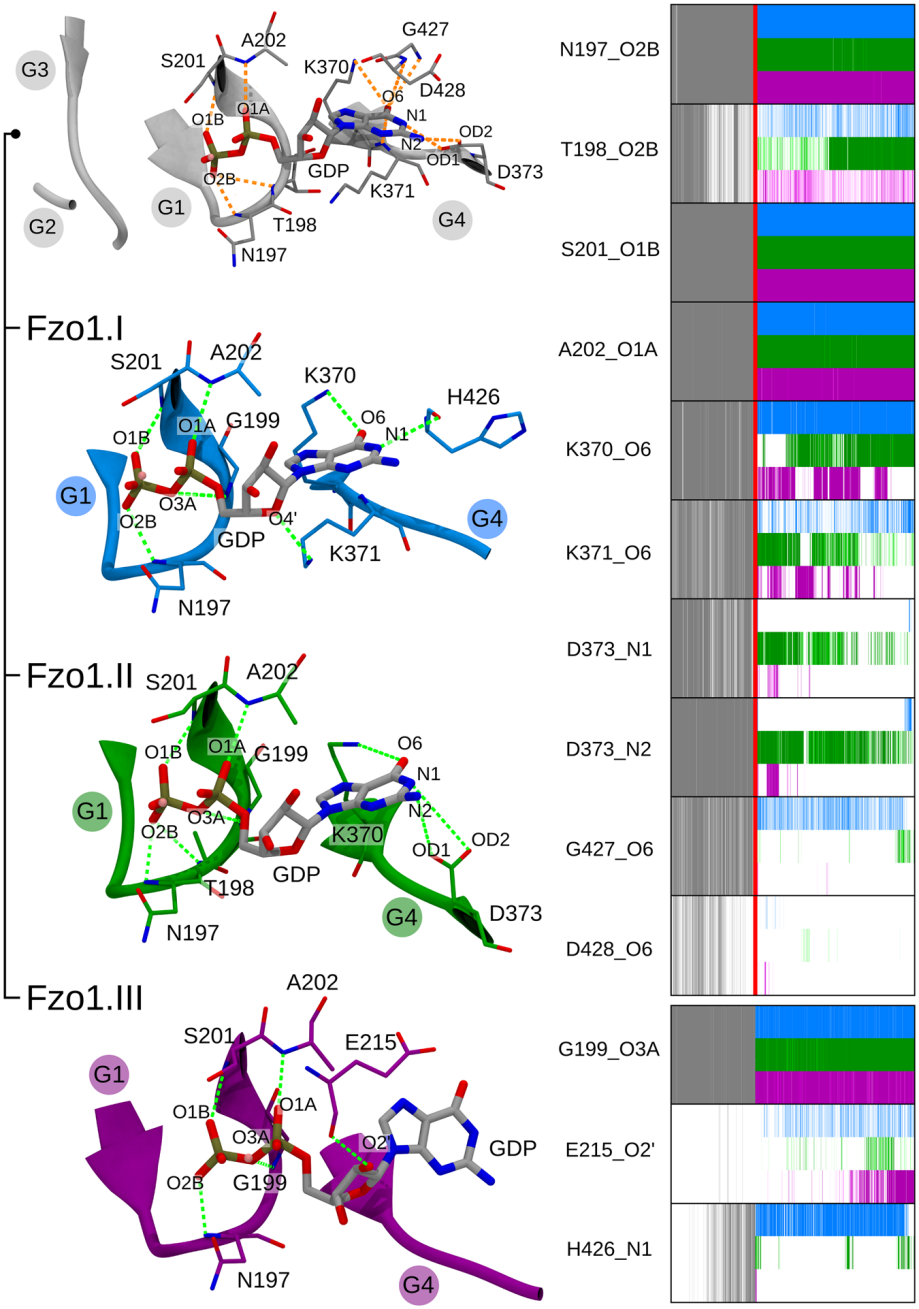
Analysis of the key protein-GDP interactions in the Fzo1 nucleotide-binding site. The colour code is *grey* during the equilibration phase, *cyan* for simulation Fzo1.I, *green* for Fzo1.II and *purple* for Fzo1.III. Distance restraints and persistent H-bonds over 50% of the simulation time are indicated with *orange* and *green* dotted lines, respectively. The G-boxes from G1 to G4 are highlighted. The evolution of the donor-acceptor distance network for the residues contacting the ligand is presented on the right. The last three blocks show the new interactions observed in Fzo1 with respect to BDLP, and the *red* line delimits the end of the equilibration phase. Donor-acceptor distances are depicted using a colour gradient from the darkest (below or equal to 3.5 Å) to lightest colours (up to 4.0 Å).

Protein-GDP interactions were modelled based on the homologous interacting residues in the BDLP template. Ten H-bonds were identified in the BDLP structure (Supplementary Table 9). The 10 corresponding donor-acceptor distances were restrained during the modelling procedure and the equilibration phase (Fig. 8 and methods for details). The time series of these distances in the three simulations were monitored during the equilibration phase and for each trajectory (Fig. 8 right and Supplementary Fig. 13).

The residues forming persistent, tight contacts were Asn197, Ser201 and Ala202. Contacts showing more variation after the restrained equilibration phase were formed by Thr198, Lys370, Lys371 and Asp373. Contacts involving Asp428 and Gly427 were predicted based on the template but were not retained in the unrestrained trajectories. An equivalent network involving the homologous residues N237 (Fzo1-K370), D240 (Fzo1-D373) and others (Supplementary Table 10) was found in the recent crystal structures from human Mfn1^18, 19^, and is conserved in our Mfn1-based model (Supplementary Fig. 10). Our approach thus provides insight into the residues that promote the stabilization of the nucleotide within the Fzo1 GTPase domain.

MD simulations further revealed new interactions in the Fzo1 model. In particular, His426 contacted the GDP.N1 in Fzo1.I after the displacement of the Asp428 residue (Fig. 8 right). Furthermore, the Gly199 residue contacted the bridging oxygen between the two phosphates (GDP.O3A), and the Glu215 residue was involved in an interaction, characterized by a 46% persistence in Fzo1.III, with the ribose in position O2′ oriented towards the backbone (Fig. 8 left). Our model also includes a magnesium ion in the nucleotide binding site, which participates in coordinating GDP with the Ser201 residue (see Supplementary Fig. 14), similar to human dynamin or atlastin^40, 41^. This feature is not only in agreement with the structure of the human Mfn1 fragment in which the Ser89 residue directly coordinates a bound magnesium ion^19^ but is also consistent with the requirement for this ion in the tethering of proteoliposomes by the Mfn1 construct^18^.

GDP primarily interacts with the G1 and G4 motifs. In particular, G1 (the P-loop) anchors both phosphate groups (Fig. 8 left). The G2 (switch I) and G3 motifs point away from the active site, suggesting a more structural role. This observation is consistent with the orientation of Thr103 in the BDLP crystal structure away from the active site^16^ and the critical role of the corresponding conserved Thr221 (*i.e*., G2 motif) in Fzo1 function^9, 11, 13^. These properties are typical of GTPase domains^41, 42^.

#### A positive charge at position 200 is essential for GTPase domain integrity

**Figure 9:**
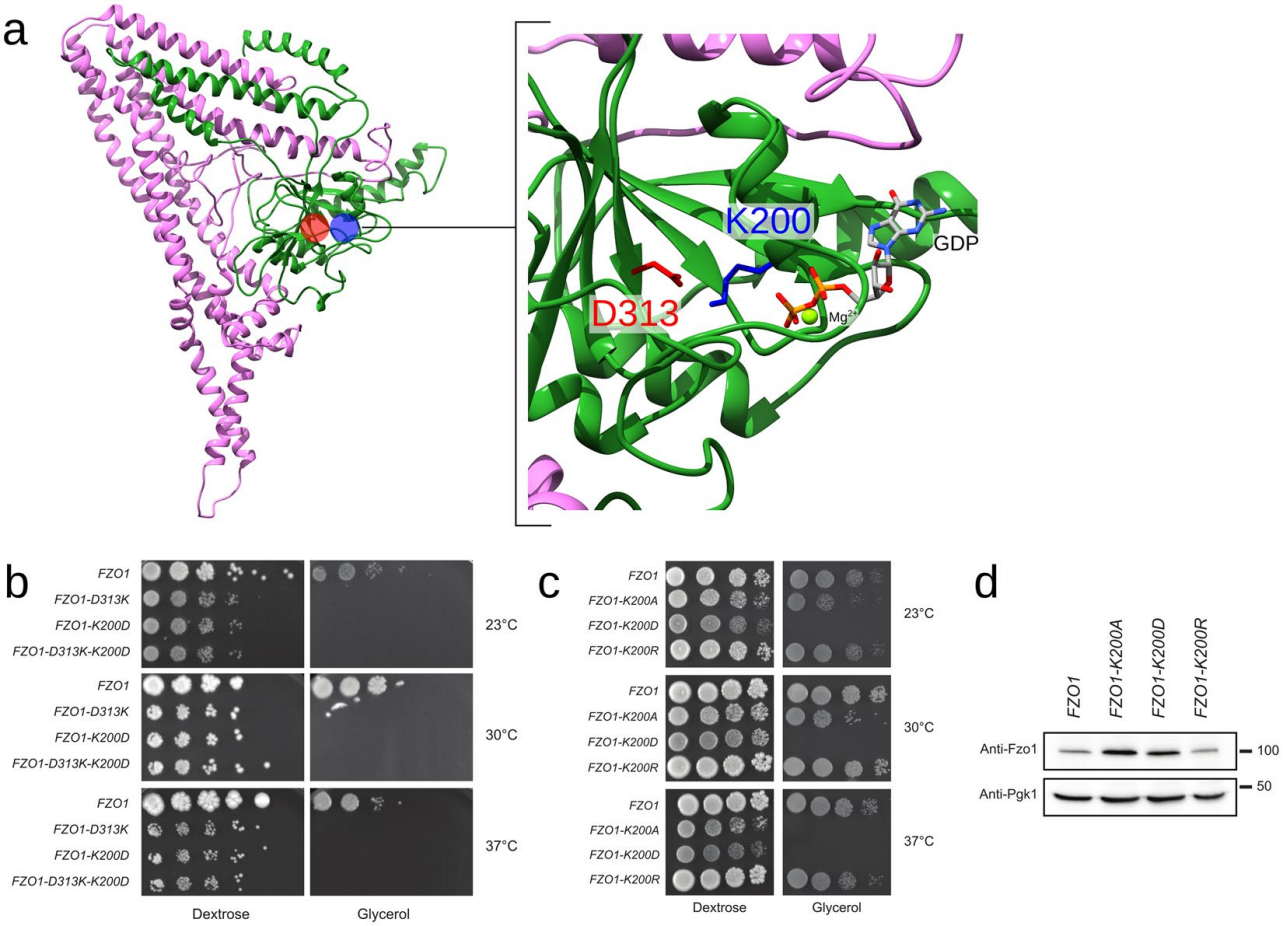
Swap mutations across the predicted salt bridge K200-D313. (**a**) Location of D313 (*red*) and K200 (*blue*) in the Fzo1 model. The most representative structure after the cluster analysis on the Fzo1.I trajectory is shown (*i.e*., the centroid). The *green* and *pink* regions correspond to the N- and C-terminal halves, respectively (Supplementary Fig. 9). The GDP atoms and the bound magnesium are indicated. (**b**) Dextrose and glycerol growth spot assay. Single mutations across the predicted interaction cause a severe respiration defect, and the double mutant (charge swap) does not rescue these defects. (**c**) Dextrose and glycerol growth spot assay. The *K200A* and *K200D* point mutations affect respiration, whereas the *K200R* mutation does not impact growth on glycerol media. (**d**) Anti-Fzo1 immunoblot of whole-cell extracts prepared from the strains used in (**c**). The Pgk1 immunoblot was used as a loading control. Molecular weight markers are indicated on the right.

The vicinity between the residues of the GTPase binding site in the model features a high confidence salt bridge that connects the G1 (Lys200) and G3 motifs (Asp313). This salt bridge is not only conserved in BDLP (Lys82 and Asp180, respectively) but also detected in all three MD replicates, Fzo1.I, Fzo1.II and Fzo1.III, with a persistence greater than 90%. Surprisingly, the charge swap strategy applied to this predicted salt bridge revealed that although the single mutations K200D and D313K induced an expected and strong inhibition of respiratory growth, the double K200D-D313K mutation (charge swap) did not rescue this defect (Fig. 9b).

The conserved lysine in the G1 motif has been widely studied using alanine/threonine mutants and has been classified as essential for mitofusin^5, 9, 11, 43^ and BDLP function^16^. In addition, the *K200A* mutant abrogates the Mdm30-dependent degradation of Fzo1 in yeast^13^. More recently, the *K88A* mutation of the corresponding lysine residue in Mfn1 was shown to promote the coordination of the γ-phosphate of GTP by Lys99, thereby inducing the drastic bending of GTP and the inhibition of hydrolysis^18^ (Supplementary Fig. 10). Moreover, the homologous position in human dynamin (Lys44) was proposed to counteract the developing negative charge of the γ-phosphate group that is released during GTP hydrolysis^41^. Thus, we reasoned that the positive charge provided by the lysine at position 200 may be essential for GTP hydrolysis, which could explain the failure of the *K200D-D313K* swap mutation to restore functional mitochondrial fusion. Furthermore, a negative charge at position 200 may weaken (if not abolish) the interaction with the nearby negatively charged nucleotide.

We introduced a conservative charged mutation to arginine at position 200 (*K200R*) and compared the effects of the *K200A*, *K200D* and *K200R* mutations on respiratory growth to test this hypothesis. Remarkably, although *K200D* abolished growth on glycerol media and *K200A* induced strong thermosensitive defects, *K200R* maintained full respiratory growth capacity at all tested temperatures (Fig. 9c). Consistent with this observation, the Mdm30-mediated degradation, and thus the GTPase domain integrity^13^, were affected in Fzo1 *K200A* and Fzo1 *K200D* but not Fzo1 *K200R* (Fig. 9d). Therefore, a positive charge in position 200 is required for Fzo1 function. This finding explains the failure of the *D313K-K200D* charge swap mutant to rescue respiratory growth but also suggests that the positive charge at position 200 participates in GTP hydrolysis.

## Conclusions

Using the homology modelling approach based on the structure of the bacterial dynamin BDLP, we have modelled the structure of the Fzo1 mitofusin in complex with the nucleotide GDP. The structural model is stable under simulation, experimentally validated *in vivo* and consistent with published data. The model provides insights at the residue level and describes Fzo1 as being partitioned into distinct structural regions: the GTPase domain, a four-helix bundle, a hinge region, and a three-helix trunk connected to the TM domain. Little is known about the precise sequence of events that may couple GTP hydrolysis to potential conformational rearrangements within mitofusins. In this regard, the conserved four-helix bundle observed in the Mfn1 partial structures^18, 19^ and in our full-length Fzo1 model is reminiscent of the characteristic bundle signalling element (BSE) of dynamins^41, 42^. The BSE domain has been proposed to transfer conformational information from the GTPase domain to the trunk region of dynamin during membrane fission catalysis^41^. Considering the continuity between the four-helix bundle and the putative hinge region in Fzo1 (Supplementary Fig. 10), this structural motif may play a similar role during membrane fusion. When comparing the Mfn1- and the BDLP-based models we have constructed, the bundles are arranged differently (Supplementary Fig. 10). While this difference may involve the artificial linker that allowed obtaining the Mfn1 crystals^18, 19^, our BDLP-based model suggests that the bundle likely adopts a distinct conformation that could provide optimal flexibility in the hinge 1a/1b regions, which warrants further insights on the structure of full-length mitofusins. Mitofusins do not function as monomers but oligomerize in both *cis* (on the same lipid bilayer) and *trans* (from opposing lipid bilayers) to mediate membrane attachment and fusion^4, 11, 19, 36, 37, 44^. As the precise principles for these oligomerization properties emerge, future studies should aim to model these complexes in presumably “active” and “inactive” conformations and thereby further elucidate the precise events that lead to mitofusin-mediated membrane fusion.

## Materials and Methods

### Bioinformatic analysis

#### Sequences identification and alignments

The *S. cerevisiae* Fzo1 target sequence was obtained from the UniProtKB database (Universal Protein Resource Knowledgebase^45^), entry: P38297. A putative template was identified using the BLASTP tool (Basic Local Alignment Search Tool for proteins^46^) against the PDB (Protein Data Bank^47^, with default settings. Homologues for the template in the phylum Cyanobacteria were then selected using the BLAST method (with default settings), from which a set of 43 sequences annotated as dynamin-like proteins was selected. The identity ranged from 22% to 87%, and the sequence coverage was greater than 65%. This set was then aligned with Expresso^48^ using structural information from the template structure with PDB-id 2J68^16^. After removing the first 100 N-terminal residues of Fzo1, we merged the multiple sequence alignment (MSA) described above with the target sequence using M-Coffee^49^. We used NCBI HomoloGene (https://www.ncbi.nlm.nih.gov/homologene, accessed 10-05-2016) to add a restricted set of 11 homologous sequences belonging to the families of fuzzy onions (FZO1), FZO-like (FZL), mitofusin (MFN1 and MFN2) (hgid: 31469, 95893, 11481 and 8915, respectively), which are listed in Supplementary Table 1.

Supplemental alignments were constructed to identify the target-template correlation in the N-terminal region. Two different alignment algorithms, Clustal Omega^50^ and T-Coffee^51^, were chosen to combine the set of 43 homologous template sequences. Then, the resulting MSA was merged with the target Fzo1 sequence with and without its first 100 N-terminal residues using M-Coffee (Supplementary Figs 1–4).

The T-Coffee method was used to align protein sequences from all the mitofusins belonging to the FZO1 family listed in Supplementary Table 1 that were used in this study.

#### Structure prediction and model assessment

The secondary structure of Fzo1 was predicted from its sequence using the following algorithms: CONCORD^52^; PSIPRED (v 3.3)^53^; PSSpred^54^; and PORTER^55^. The LigPlot+ program^56^ was used to inspect the homologous residues in the target and template that contact the GDP substrate using a donor-acceptor distance of 3.5 Å. The PCOILS v1.0.1 algorithm^57^ was used to predict heptad periodicity in Fzo1 and BDLP. Disordered residues in the target sequence were investigated using the predictor DISOPRED3^25^.

#### Transmembrane domain

The BDLP template used for modelling Fzo1 possesses a hydrophobic region (res 572–606, Supplementary Fig. 8) that mediates lipid binding but is not a TM domain^16^. As a result, the predicted TM helices of Fzo1 (Supplementary Table 11) do not match in the final target-template alignment after refinement (Supplementary Fig. 11). For this reason, the *ab initio* prediction of the helix dimer structure was crucial in the modelling process.

The location of the TM segments was identified using the predictors MEMSAT-SVM^58^, OCTOPUS^59^ and TMpred^60^ (Supplementary Table 11). *Ab initio* modelling of the TM segments was performed using the PREDDIMER server^27^, and the best model was selected according to the proposed ranking score (*F*_*SCOR*_ 3.113).

### Strategy used to model the Fzo1-GDP complex

We manually refined the target-template alignment described above to avoid fragmenting the secondary structure elements. In particular, we modified the alignment to open a gap of 21 positions within the predicted TM helices of Fzo1. This modification enabled us to reconstitute the predicted fold for the C-terminal fragment (res 826–855) that was previously shown to be required for mitochondrial respiration^34^, but was unstructured and located outside the template in the initial alignment. Consistently, this modification positioned the residue L819, which induces a severe loss of function (LOF) when mutated to proline^11^, from a nearby unstructured region precisely within the putative hinge thought to enable the GTPase domain-dependent rearrangements. Moreover, consistent with the crystal structure of human Mfn1, this C-terminal fragment may participate in stabilizing the identified four-helix bundle^18, 19^. Finally, guided by the secondary structure predictors, small adjustments were made to restore helices that were disrupted by insertions and deletions (indels).

We combined three different templates to model the Fzo1 structure. The TM segments (res 706–726 and 737–757) were based on the *ab initio* model, the pentapeptide 216–220, which was unresolved in structure 2J68, was modelled on residues 98–102 from structure 2J69 (3.0 Å^16^), and the remainder of the protein followed structure 2J68. One hundred models were generated using the MODELLER program (v 9.15)^61^. The GDP nucleotide coordinates from structure 2J68 were included during the modelling procedure. During modelling, harmonic restraints were imposed on donor-acceptor distances, based on the LigPlot+ analysis of protein-nucleotide interactions in template 2J68. Moreover, we imposed canonical α-helix conformations for residues 101–123, 299–304, 426–433, 548–553, 700–726, 737–757 and 847–855, as well as β-strand conformations for residues 234–241 and 281–283.

We performed loop refinement of the intermembrane loop (res 727–736) and we applied the loop modelling routine of MODELLER^62^ resulting in fewer Ramachandran outliers. We ranked solutions according to the discrete optimized protein energy (DOPE) method^63^.

The Dunbrack rotamer library^64^ implemented in University of California, San Francisco (UCSF) Chimera^24^ was used to change the rotamers of the Lys727, Lys735 and Lys736 residues, which are located within the intermembrane loop, by orienting the charged side-chains towards water to stabilize the TM domain during the MD simulation. Similarly, the rotamer of residue Ile753 was changed to increase TM helix packing. The PROPKA method^65^ (v 3.00) implemented in the automated PDB2PQR pipeline^66^ (v 2.0.0) was used to assign protonation states at pH 7. A double protonation was predicted for the His240 residue. The lipid-buried Lys716 residue is conserved in half of the genes belonging to the FZO1 family (Supplementary Table 1 and Supplementary Fig. 15). According to recent data, lysine residues are titrated in a membrane environment, suggesting that changes in the ionization state may regulate membrane-protein function^67^. Therefore, the protonation state of the lipid-buried Lys716 was manually set to a neutral amine. The stereochemical quality was assessed using the MolProbity server^22^ and PROCHECK^23^. The model obtained was then oriented with respect to the lipid bilayer using the PPM server^68^. These coordinates were subsequently manually adjusted by a 6° rotation around the *x* axis and a translation of −1.5 Å along the *z* axis to improve the lipid-packing around the Fzo1 TM domain, and empty spaces in the bilayer were avoided during model building using the CHARMM-GUI pipeline (see below).

### Simulation setup and equilibration

The GROMACS 5.0.4 program was used to perform the MD simulations^69^. The simulation system was assembled using the CHARMM-GUI Membrane Builder^70^ with the CHARMM36 force field^71^. In order to mimic the mitochondrial outer membrane composition, the protein model was embedded in a 1:1 mixed lipid bilayer composed of 156 molecules of palmitoyl-oleoyl-phosphatidylcholine (POPC) and 156 molecules of palmitoyl-oleoyl-phosphatidylethanolamine (POPE). The solvent is composed of a 150 mM KCl solution with 36,919 TIP3P water molecules^72^. One magnesium ion was manually positioned between the α and β GDP phosphates, consistent with crystal structures 2X2E, 3T34, 3W6P and 5CA9 for human dynamin 1^41^, dynamin-related protein 1 A from *Arabidopsis thaliana* ^73^, human dynamin-1-like protein^74^ and Sey1p from *Candida albicans* ^75^, respectively. Two K^+^ counterions were subsequently added to neutralize the system. Supplementary Table 12 reports the composition of the simulation system. Before running the simulations, the system was subjected to 8000 steps of energy minimization. Afterwards, the structure was thermalized at 310 K for 200 ps using the NVT ensemble, with a time step of 2 fs and position restraints of 1000 kJ·mol^−1^·nm^−2^ on all protein heavy atoms. Subsequently, the same restraint settings were applied in 10 ns simulations using the NPT ensemble to allow the lipids to relax. The optimized and relaxed system was further equilibrated for a total of 250 ns using the NPT ensemble by combining position restraints on side-chains and backbone atoms, which were gradually relaxed from 1000 to 5 kJ·mol^−1^·nm^−2^ in seven successive steps. Simultaneously, we maintained distance restraints of 1000 kJ·mol^−1^·nm^−2^ for the previously identified GDP homologous hydrogen bond network (see above). After 250 ns of equilibration, three replicas of the same system (Fzo1.I, Fzo1.II and Fzo1.III) were simulated for a total of 500 ns with an integration time step of 2 fs in the NPT ensemble under periodic boundary conditions. Long-range electrostatics were managed using the particle-mesh Ewald (PME) method^76^. All bond lengths were constrained using the LINCS algorithm^77^. Global translational and rotational motions were removed every 100 steps. Atomic positions were saved every 50 ps, and the first 100 ns were not used for the analysis.

### Trajectory analysis and molecular graphics

Root mean-square deviation (RMSD) and fluctuation (RMSF) analyses were performed using tools from the GROMACS package (g_rmsf and g_rmsf, respectively). A refined RMSD value was computed by omitting residues that fulfilled one or more of the following criteria: when a residue possesses a large RMSF value, is less conserved, unstructured, predicted to be disordered, hypothesized to be implicated in putative conformational change (*e.g*., hinge regions), unresolved in the template, and involved in a progressive loss of folding.

Secondary structure elements were identified using the do_dssp tool implemented in GROMACS based on the DSSP method^78^. Hydrogen bonds and salt bridges were identified using g_hbond from GROMACS and VMD 1.9.2^79^ (http://www.ks.uiuc.edu/Research/vmd/), respectively. Contacts within a distance cut-off of 3.5 Å (and up to 30 degree off-axis angle) and 6.5 Å were considered for H-bonds and salt bridges, respectively, to assess these interactions. Other analyses were conducted using in-house programs. When not otherwise specified, the persistence means how much the considered event occurs during the simulation time, expressed as a percentage over the number of frames. The cluster analysis was performed using g_cluster from GROMACS with the GROMOS method^80^, with a cut-off of 0.25 Å. Molecular graphics were generated with the VMD 1.9.2^79^ (http://www.ks.uiuc.edu/Research/vmd/) and UCSF Chimera 1.9^24^ packages. Data were plotted using Grace (http://plasma-gate.weizmann.ac.il/Grace/).

### Plasmids, yeast strains and growth conditions

FZO1 mutant plasmids are listed in Supplementary Table 13. Plasmids MC377 to MC382, MC396 and MC397 were synthesized by GeneCust (Ellange, Luxembourg). A QuikChange Lightning Site-Directed Mutagenesis Kit (Agilent Technologies, Santa Clara, California, USA) was used to generate plasmids MC400 and MC406 to MC408. Mutagenic primers were designed based on the MC250 plasmid sequence with the web-based QuikChange Primer Design Program available online at www.agilent.com/genomics/qcpd.

Yeast strains are listed in Supplementary Table 14. Standard methods were used for growth, transformation and genetic manipulation of *S. cerevisiae*. Minimal synthetic media [Difco yeast nitrogen base (Voigt Global Distribution, Inc., Lawrence, Kansas, USA), and drop-out solution] supplemented with 2% dextrose (SD) or 2% glycerol (SG) were prepared as previously described^81^.

Strains lacking *FZO1* lose their mitochondrial DNA because of decreased mitochondrial fusion efficiency^9, 26^. Consequently, a plasmid-shuffle strategy was used as a reliable analysis of *fzo1* mutants *in vivo*. The *FZO1* shuffle strain MCY571 (*fzo1Δ* strain covered by an *FZO1* shuffling plasmid) was employed. *MDM30* was chromosomally deleted in the *FZO1* shuffle strain using a previously described method^82^ to generate the *mdm30Δ* strain (MCY585).

Shuffle strains were transformed with the plasmids shown in Supplementary Table 13 and plated on SD selective media lacking uracil and tryptophan. Ten colonies were systematically isolated on SD selective media and replica-plated on 5′-fluoroorotic acid (5′-FOA) plates. Strains grown on 5′-FOA plates and cured from the *FZO1* shuffling plasmid were in turn replica-plated on SD and SG selective plates lacking tryptophan. The glycerol growth phenotypes of strains cured from the shuffling plasmids were reproducibly observed in 100% of the clones tested after 1 to 3 days of growth at 30 °C. Representative clones were subsequently used for spot assays and the preparation of protein extracts.

### Spot assays

Cultures grown overnight in SD selective media lacking tryptophan were pelleted, resuspended at an optical density at 600 nm (OD_600_) of 1, and serially diluted (1:10) five times in water. Three microliters of the dilutions were spotted onto SD- and SG-selective plates and grown for 2 to 4 days (dextrose) or 3 to 6 days (glycerol) at 23 °C, 30 °C or 37 °C.

### Protein extracts and immunoblotting

Cells grown in SD-selective media lacking tryptophan were collected during the exponential growth phase (OD_600_ = 0.5–1). Total protein extracts were prepared using the NaOH/trichloroacetic acid (TCA) lysis technique^83^. Proteins were separated on 8% SDS-PAGE gels and transferred to nitrocellulose membranes (Amersham^TM^ Hybond^TM^-ECL; GE Healthcare, Little Chalfont, United Kingdom). The primary antibodies used for immunoblotting were monoclonal anti-Pgk1 (Abcam, Cambridge, United Kingdom), monoclonal anti-myc (9E10), and polyclonal anti-Fzo1 (generated by GeneCust). Primary antibodies were detected using horseradish peroxidase-conjugated secondary anti-mouse or anti-rabbit antibodies (HRP, Sigma-Aldrich, Saint-Louis, Missouri, USA), followed by incubation with a Clarity^TM^ Western Kit (Bio-Rad, Hercules, California, USA). Images of the immunoblots were acquired using a Gel Doc^TM^ XR+ (Bio-Rad) and analysed using the Image Lab 3.0.1 software (Bio-Rad). The anti-Fzo1 is not sensitive enough to detect ubiquitylated species of the mitofusin. For this reason, the ubiquitylation status of Fzo1 was analyzed using *FZO1-13MYC* strains and immunoblotting with anti-myc.

## Electronic supplementary material

Supplementary material
