# Supplementary material for: A membrane-inserted structural model of the yeast mitofusin Fzo1
Source: Sci Rep. 2017 Aug 31;7:10217. doi: 10.1038/s41598-017-10687-2 (PMC5578988; doi:10.1038/s41598-017-10687-2)
Supplement: Supplementary file 1 — Supplementary material [file 41598_2017_10687_MOESM1_ESM.pdf]

A membrane-inserted structural model of the yeast mitofusin Fzo1

De Vecchis, D.[1], Cavellini, L.[2], Baaden, M.[1], Hénin, J.[1], Cohen, M.M.[2], and Taly, A[1].

Institut de Biologie Physico-Chimique, Centre National de la Recherche Scientifique, Paris, France.

[1]Laboratoire de Biochimie Théorique, UPR 9080.

[2]Laboratoire de Biologie Cellulaire et Moléculaire des Eucaryotes, UMR 8226.

Supplementary Table 1 | Fzo1 homologue sequences considered in this study. See next page for caption.

Family	Species	Gene (Id)	Length	Identity (%)	Similarity (%)
MFN1 (11481)	*Homo sapiens*	MFN1 (55669)	741	19	41
	Mus musculus	Mfn1 (67414)	741	18	43
	Gallus gallus	MFN1 (424973)	740	18	40
	Pan troglodytes	MFN1 (460861)	741	18	40
	Macaca mulatta	MFN1 (709570)	741	20	42
	Canis lupus familiaris	MFN1 (488086)	742	20	42
	Bos taurus	MFN1 (515180)	742	19	42
	Rattus norvegicus	Mfn1 (192647)	741	18	42
	Xenopus tropicalis	mfn1 (548943)	738	22	43
	Danio rerio	Mfn1b (393620)	740	21	43
MFN2 (8915)	*Homo sapiens*	MFN2 (9927)	757	17	41
	Danio rerio	mfn2 (567448)	757	18	41
	Caenorhabditis elegans	fzo-1 (173990)	774	22	40
	Drosophila melanogaster	Marf (31581)	810	21	42
	Xenopus tropicalis	mfn2 (549268)	756	19	42
	Pan troglodytes	MFN2 (457958)	757	17	41
	Macaca mulatta	MFN2 (100427191)	634	19	43
	Canis lupus familiaris	MFN2 (487439)	757	17	41
	Bos taurus	MFN2 (534574)	757	17	40
	Mus musculus	Mfn2 (170731)	757	17	41
	Rattus norvegicus	Mfn2 (64476)	757	18	41
	Rattus norvegicus	LOC100911485 (100911485)	757	18	41
	Gallus gallus	MFN2 (419484)	807	18	42
	Anopheles gambiae	AgaP_AGAP001802 (1281313)	776	18	40
FZL (95893)	*Arabidopsis thaliana*	FZL (839566)	912	23	44
	Oryza sativa	Os05g0390100 (4338678)	803	25	45
FZO1 (31469)	*Saccharomyces cerevisiae*	FZO1 (852477)	855	20	43
	Kluyveromyces lactis	KLLA0E24179g (2894339)	825	19	40
	Schizosaccharomyces pombe	Fzo1 (2539859)	758	20	44
	Eremothecium gossypii	AGOS_ABR195C (4619254)	808	20	42
	Neurospora crassa	NCU00436 (3872838)	918	19	41
	Magnaporthe oryzae	MGG_05209 (2675586)	911	19	40

Supplementary Table 1 | Fzo1 homologue sequences considered in this study. The source for homologue sequences is the NCBI HomoloGene tool (https://www.ncbi.nlm.nih.gov/homologene, accessed 10-05-2016). The species highlighted by a gray background are those considered in the final target-template alignment. The family name and the HomoloGene identifier are indicated. MFN1, Mitofusin 1, MFN2, Mitofusin 2, FZL, FZO-like and FZO1, fuzzy onions. The identity and similarity with respect to the template BDLP, were obtained by T-Coffee using the *slow_pair* method which is recommended when the sequences are distantly related [47]. The BLOSUM45 matrix was used for the similarity score.

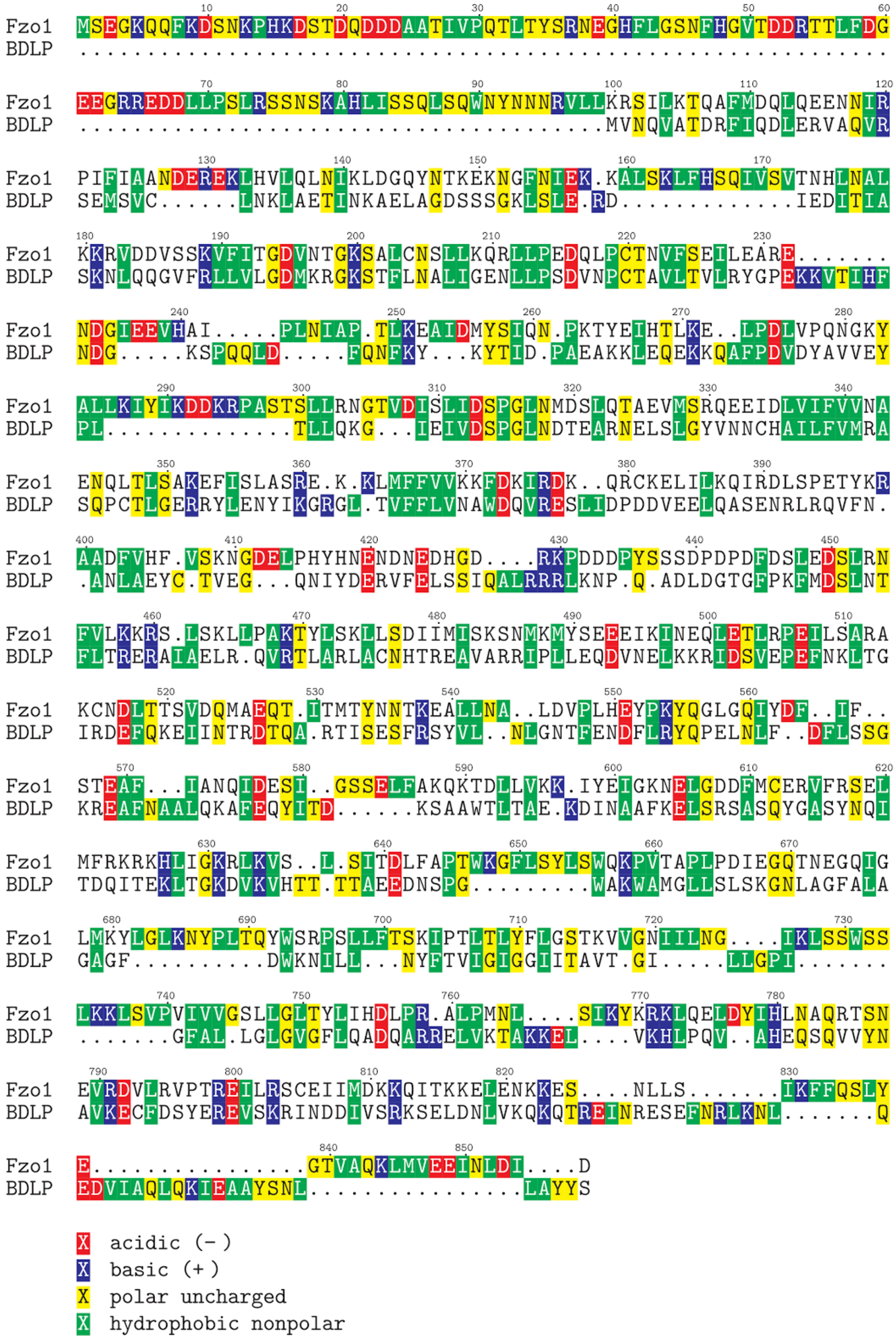

Supplementary Figure 1 | Target-template alignment using the Clustal Omega method considering the whole sequences. The set of 43 sequences from the cyanobacteria (see Methods) were aligned using Clustal Omega [46]. Subsequently, the generated multiple alignment was merged with the sequence from the target Fzo1 using M-Coffee[45].

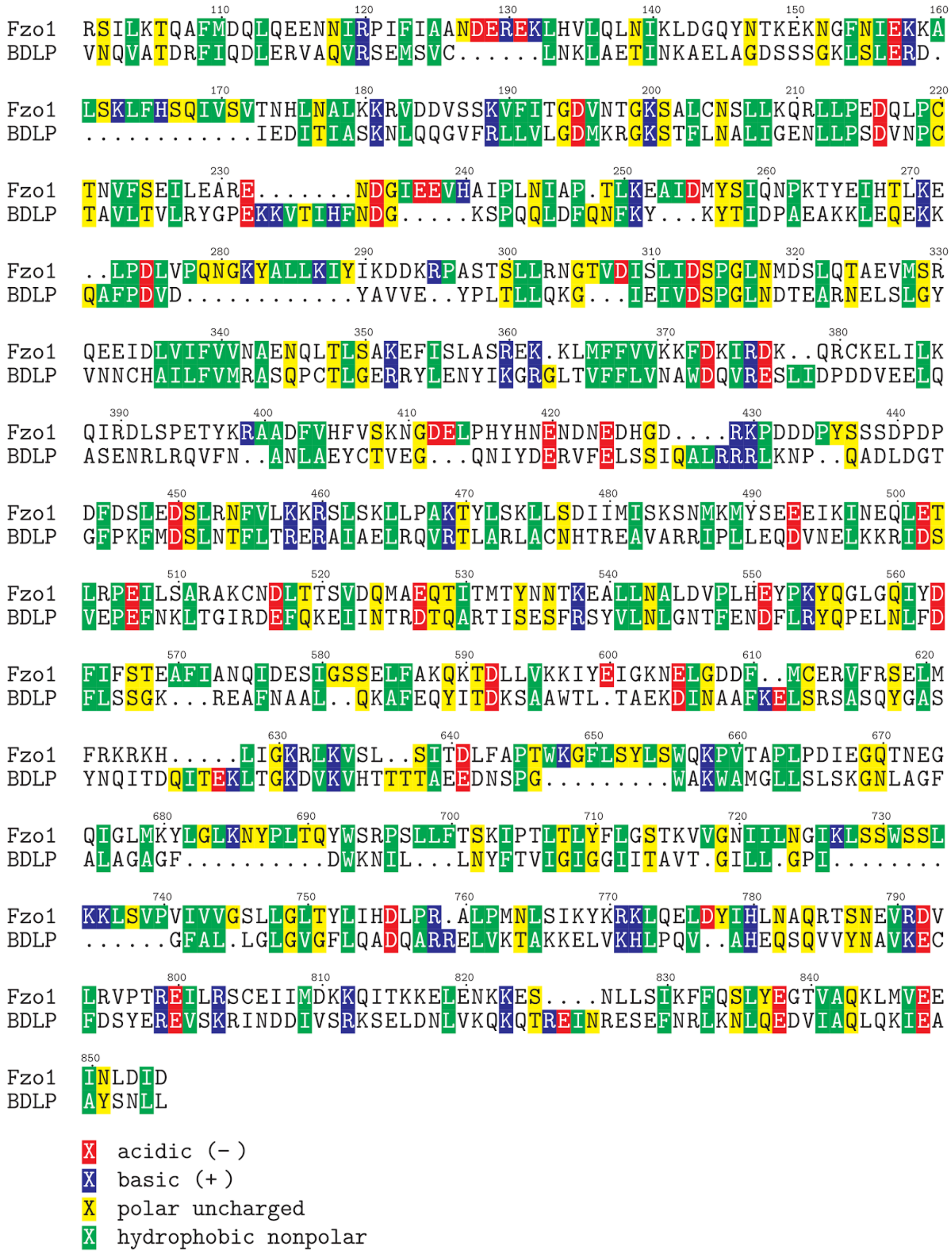

Supplementary Figure 2 | Target-template alignment using the Clustal Omega method without the first one hundred N-terminal residues from Fzo1. The set of 43 sequences from the cyanobacteria (see Methods) were aligned using Clustal Omega [46]. Subsequently, the generated multiple alignment was merged with the sequence from the target Fzo1 without its first one hundred N-terminal residues, using M-Coffee [45].

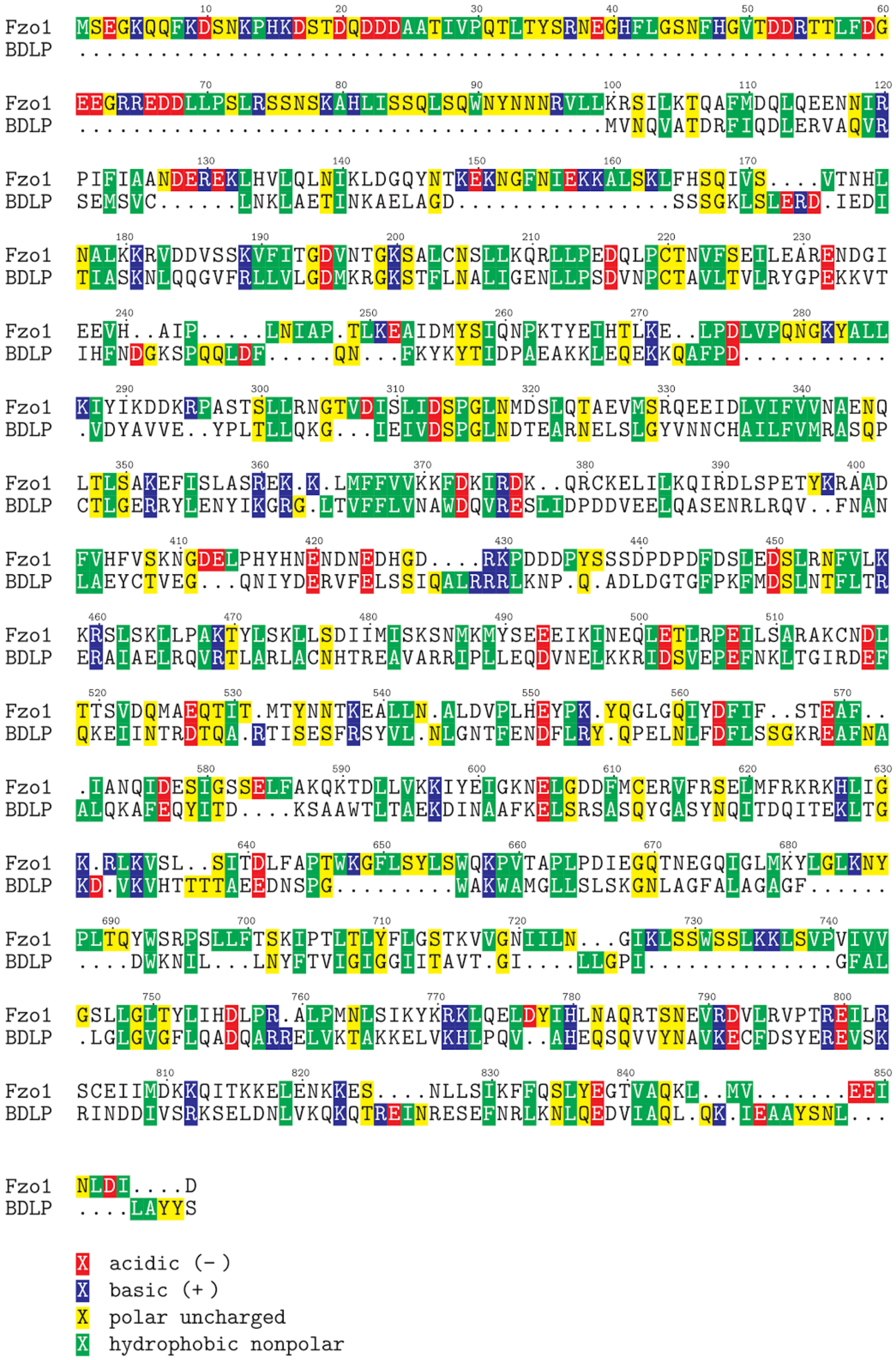

Supplementary Figure 3 | Target-template alignment using T-coffee considering the whole sequences. The set of 43 sequences from the cyanobacteria (see Method) were aligned using T-coffee [47]. Subsequently, the generated multiple alignment was merged with the sequence from the target Fzo1, using M-Coffee [45].

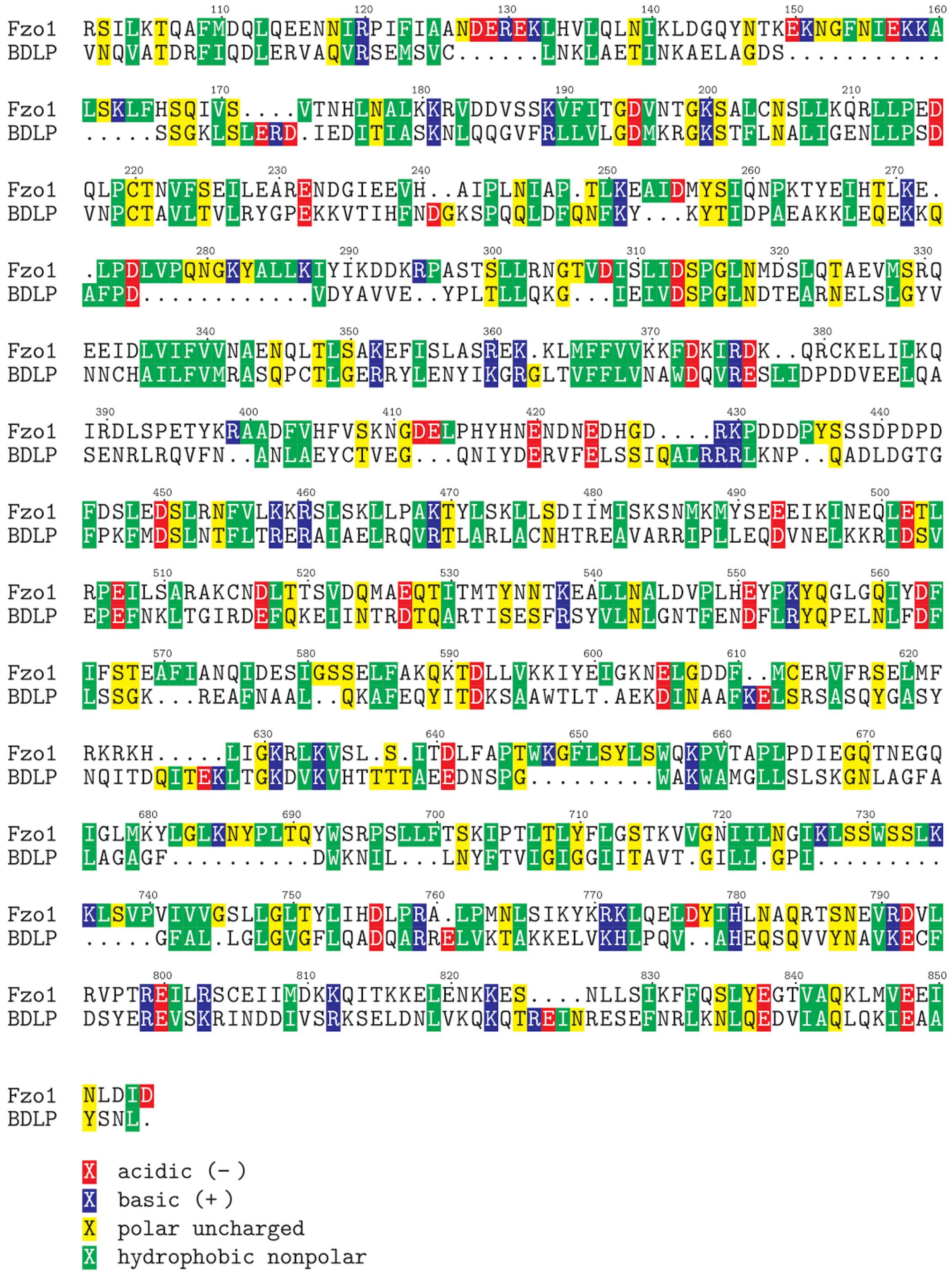

Supplementary Figure 4 | Target-template alignment using T-coffee without the first one hundred N-terminal residues from Fzo1. The set of 43 sequences from the cyanobacteria (see Methods) were aligned using T-coffee [47]. Subsequently, the generated multiple alignment was merged with the sequence from the target Fzo1 without its first one hundred N-terminal residues, using M-Coffee [45].

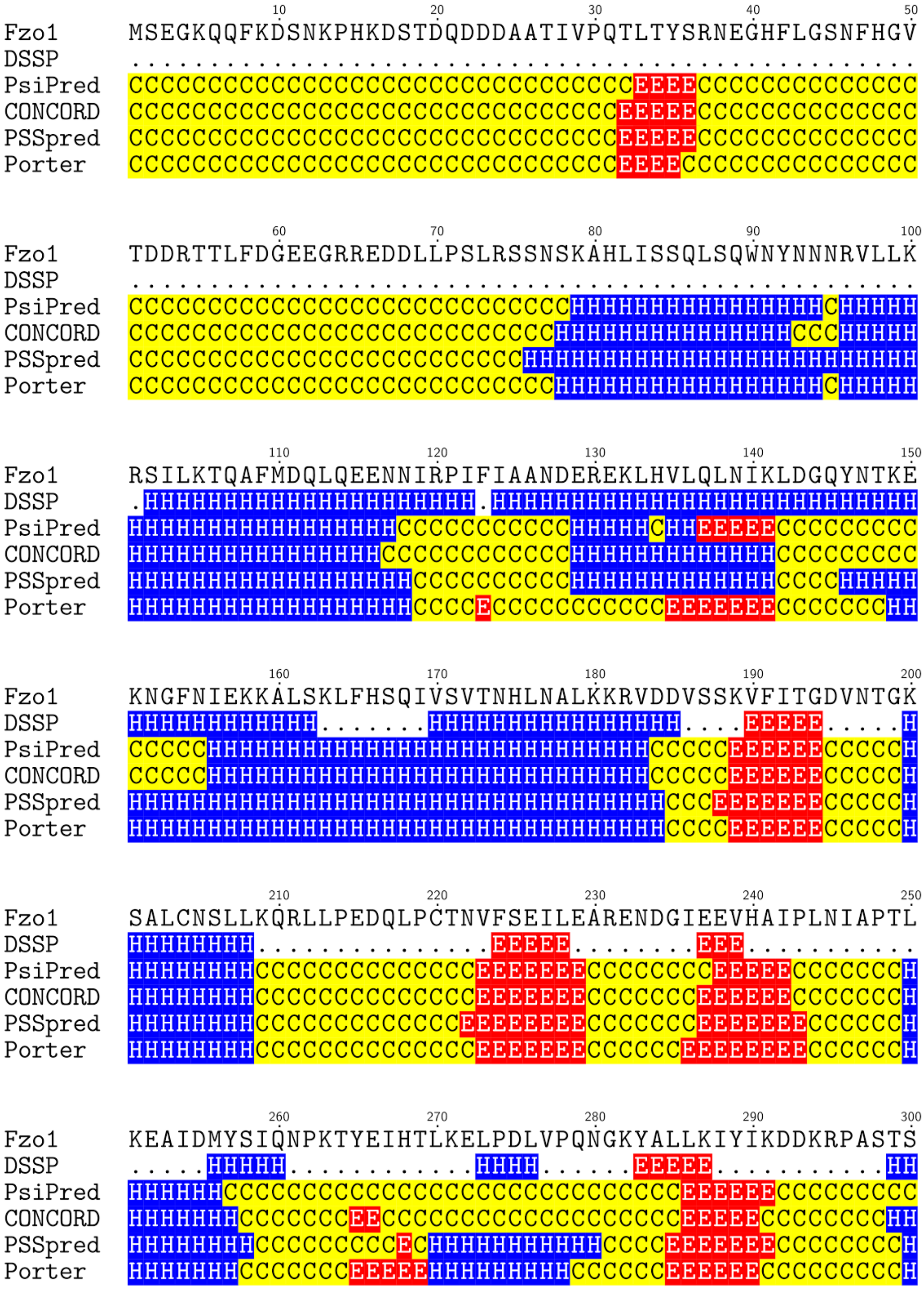

Supplementary Figure 5 | Secondary structure prediction for Fzo1. Continue next page.

```
                               310       320       330       340       350
Fzo1     LLRNGTVDISLIDSPGLNMDSLQTAEVMSRQEEIDLVIFVVNAENQLTLS
DSSP     HHHH......EEE.....HHHHHHHHHH.HHHH..EEEEEEE......HH
PsiPred  CCCCCCCCEEEECCCCCCCCCCCHHHHHHHHHCCCEEEEEEECCCCCCHH
CONCORD  HHCCCCCCEEEEECCCCCCCCHHHHHHHHHHHCCCEEEEEEECCCCCCHH
PSSpred  HHHCCCCEEEEEECCCCCCCHHHHHHHHHHHCCCCEEEEEECCCCCCCHH
Porter   HHHCCCEEEEEEECCCCCCCCHHHHHHHHHHHCCCEEEEEEECCCCCCHH

                               360       370       380       390       400
Fzo1     AKEFISLASREKKLMFFVVKKFDKIRDKQRCKELILKQIRDLSPETYKRA
DSSP     HHHH..........EEEEEE....HHHH......HHHHHHHHHHHHHHHH
PsiPred  HHHHHHHHHHCCCCEEEEEECCCCCCCHHHHHHHHHHHHHHHCHHHHHHC
CONCORD  HHHHHHHHHHCCCCEEEEEECCCCCCCHHHHHHHHHHHHHHHCCHHHHHH
PSSpred  HHHHHHHHHHHCCCEEEEEECCCCCCCHHHHHHHHHHHHHHHHHHHCCCC
Porter   HHHHHHHHHHCCCCEEEEECCHHCCCCHHHHHHHHHHHHHHCCCCHHHHH

                               410       420       430       440       450
Fzo1     ADFVHFVSKNGDELPHYHNENDNEDHGDRKPDDDPYSSSDPDPDFDSLED
DSSP     HHH..............HHHHEEE....HHHHHHHH........HHHHHH
PsiPred  CCEEEEECCCCCCCCCCCCCCCCCCCCCCCCCCCCCCCCCCCCCHHHHHH
CONCORD  CCCEEEEECCCCCCCCCCCCCCCCCCCCCCCCCCCCCCCCCCCCHHHHHH
PSSpred  CCEEEEEECHHHCCCCCCCCCCCHHHHHHHCCCCCHHHHHHCCCHHHHHH
Porter   HHCEEEEECCCCCCCCCCCCCCCCCCCCCCCCCCCCCCCCCCCCHHHHHH

                               460       470       480       490       500
Fzo1     SLRNFVLKKRSLSKLLPAKTYLSKLLSDIIMISKSNMKMYSEEEIKINEQ
DSSP     HHH.......HHHHHHHHHHHHHHHHHHHHHHHHHHHHHHH..HHHHHHH
PsiPred  HHHHHHHHHHHHHHHCHHHHHHHHHHHHHHHHHHHHHHHHHHHHHHHHHH
CONCORD  HHHHHHHHHHHHHHHHHHHHHHHHHHHHHHHHHHHHHHHHHHHHHHHHHH
PSSpred  HHHHHHHHHHHHHHHHHHHHHHHHHHHHHHHHHHHHHHHHHHHHHHHHHH
Porter   HHHHHHHHHHHHHHHHHHHHHHHHHHHHHHHHHHHHHHHHHHHHHHHHHH

                               510       520       530       540       550
Fzo1     LETLRPEILSARAKCNDLTTSVDQMAEQTITMTYNNTKEALLNALDVPLH
DSSP     HHHHHHHHHHHHHHHHHHHHHHHHHHHHHHHHHHHHHHHHHHHH...HHH
PsiPred  HHHHHHHHHHHHHHHHHHHHHHHHHHHHHHHHHHHHHHHHHHHHHHCCCC
CONCORD  HHHHHHHHHHHHHHHHHHHHHHHHHHHHHHHHHHHHHHHHHHHHHCCCCC
PSSpred  HHHHHHHHHHHHHHHHHHHHHHHHHHHHHHHHHHHHHHHHHHHHHHHHHH
         HHHHHHHHHHHHHHHHHHHHHHHHHHHHHHHHHHHHHHHHHHHHHHHCCC

                               560       570       580       590       600
Fzo1     EYPKYQGLGQIYDFIFSTEAFIANQIDESIGSSELFAKQKTDLLVKKIYE
DSSP     HHHH..............HHHHHHHHHHH.HHHHHHHHHHHHHHHHHHHH
PsiPred  CCCCCCCCHHHHHHHHHHHHHHHHHHHHHHHHHHHHHHHHHHHHHHHHHH
CONCORD  CCCCCCCCHHHHHHHHHHHHHHHHHHHHHHHHHHHHHHHHHHHHHHHHHH
PSSpred  HCCCCCCHHHHHHHHHHHHHHHHHHHHHHHHHHHHHHHHHHHHHHHHHHH
Porter   CCCCCCCHHHHHHHHHHHHHHHHHHHHHHHHHHHHHHHHHHHHHHHHHHH
```

Supplementary Figure 5 | Secondary structure prediction for Fzo1. Continue next page.

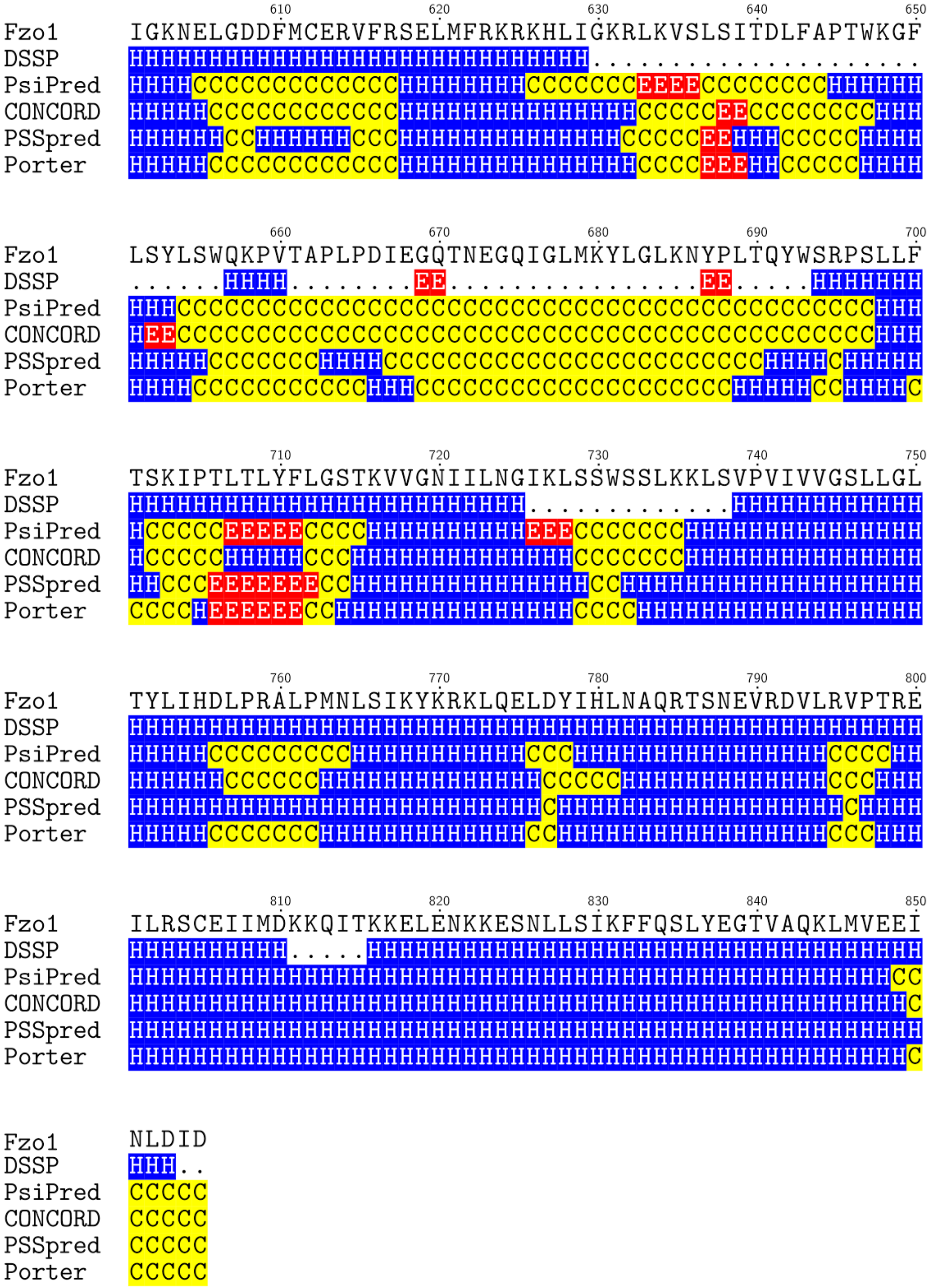

Supplementary Figure 5 | Secondary structure prediction for Fzo1. The name of each predictor used in this study is indicated and the sequence of Fzo1 is aligned to the respective output. The structure submitted to the method DSSP [77] is the structural Fzo1 model from this study after minimization. The color code is H, α-helix (*blue*); E, β-sheet (*red*) and C, coil (*yellow*). Methods used are: DSSP [77], PsiPred [49], CONCORD [48], PSSpred [50] and Porter [51].

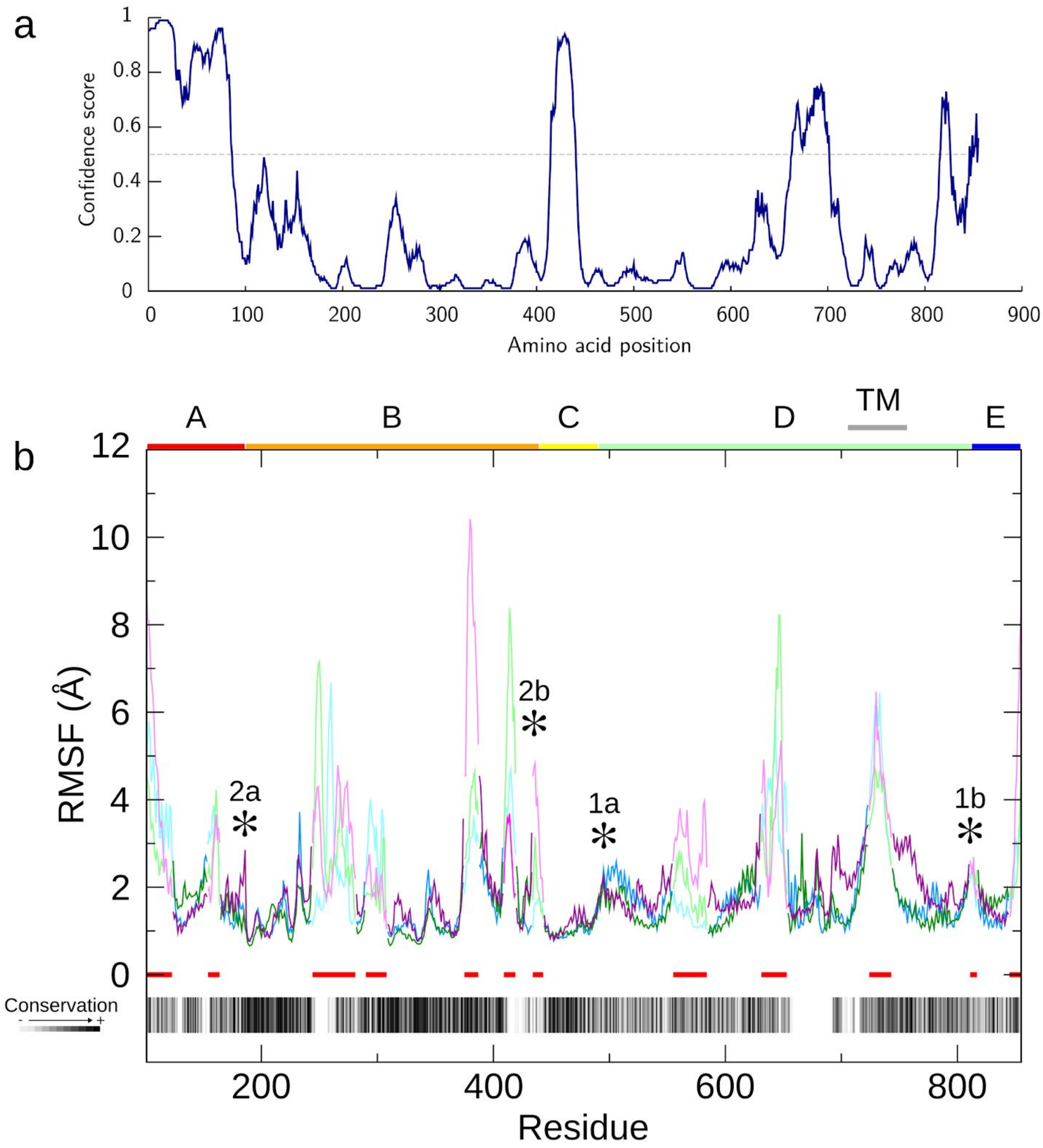

Supplementary Figure 6 | (a) Predicted disordered region in Fzo1 according the predictor DISOPRED3 [22]. The first disordered region (res 1-86) is located upstream of the N-terminal coiled-coil HRN (res 91-190). The second region (res 415-440) resides between the N- (res 1-415) and C-terminal (res 416-855) halves proposed for Fzo1 [11,13]. The third region (res 663-701) is located in the protein core, whereas a fourth one (res 816-826) is detected in the C-terminal domain, nearby the putative hinge 1b. **(b) Root-mean-square fluctuation (RMSF) per residue in all three trajectories (Fzo1.I, *cyan*; Fzo1.II, *green*; Fzo1.III, *purple*).** Positions of the putative hinges that encompass the fragments from A to E (TM, transmembrane), are highlighted with asterisks, the names being the same as proposed for the BDLP template [16]. Paler colours and *red* bars under the plot mark the unstructured/highly flexible regions. Bottom: bar plot indicating conservation per residue of the physico-chemical properties determined by Jalview [85] based on a multiple alignment (Supplementary Fig. 15) of mitofusins belonging to the family of FZO1 (Supplementary Table 1).

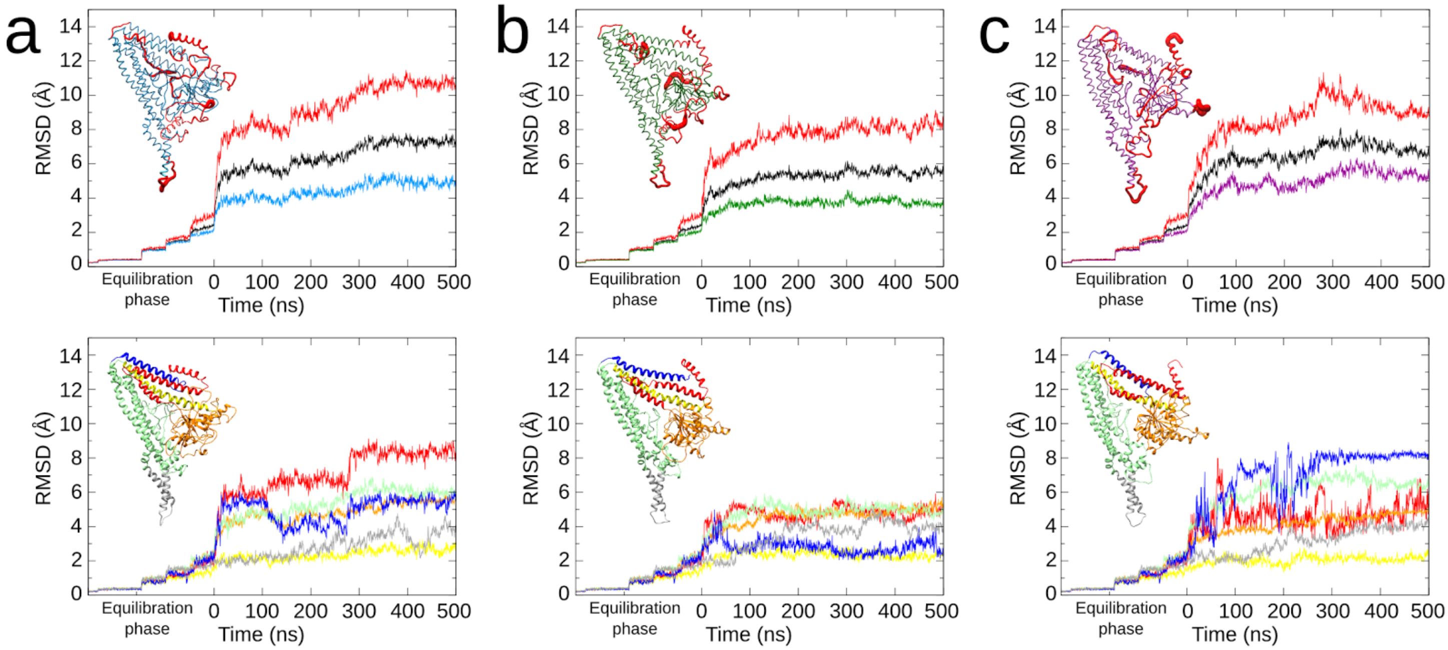

Supplementary Figure 7 | Root-mean-square deviation (RMSD) of the protein coordinates as a function of time in MD simulations. Panels (**a**), (**b**) and (**c**) are for the models Fzo1.I, *cyan*; Fzo1.II, *green* and Fzo1.III, *purple*, respectively. In the **upper panels**, the *black* line, the red and the one coloured accordingly each trajectory identify values computed considering the whole protein, the highly flexible/unstructured residues (discussed in the text) and the protein core, respectively. The **lower panels** show the RMSD per-fragment: A (*red*), B (*orange*), C (*yellow*), D (*pale green*) and E (*blue*), according to the putative hinges (see also Fig. 1a and Supplementary Table 3). The transmembrane segment is indicated in *gray*. The values are computed on the alpha carbon atoms with respect to the model after the minimization. In every plot the structure is colored accordingly and represents the last frame from the corresponding simulation time. In the upper panels the structures are depicted in tube representation of varying thickness as a function of the Root Mean-Square Fluctuation (RMSF).

Supplementary Table 2 | Root mean-square deviations.

Simulation	Mean (last frame) ± sd
Fzo1.I	4.6 (7.3) ± 2.67
Fzo1.II	3.8 (5.7) ± 2.06
Fzo1.III	4.6 (6.5) ± 2.68

The RMSDs are calculated for the Cα atoms fitting on the same set on the reference structure after the minimization. sd, standard deviation. Values are in Å.

Supplementary Table 3 | Per-fragment root mean-square deviations.

	**Fzo1.I**	**Fzo1.II**	**Fzo1.III**
**$A_{101-185}$**	5.0 (8.0) ± 3.2	3.4 (5.1) ± 1.9	3.4 (5.3) ± 1.9
**$B_{186-439}$**	3.6 (5.6) ± 1.9	3.4 (5.1) ± 1.8	3.1 (4.9) ± 1.6
**$C_{440-490}$**	1.8 (2.8) ± 0.9	1.8 (2.4) ± 0.8	1.6 (2.1) ± 0.7
**$D_{491-812}$**	3.9 (5.9) ± 2.2	3.6 (4.8) ± 1.9	4.4 (6.4) ± 2.5
**$E_{813-855}$**	3.5 (5.4) ± 2.0	2.2 (2.4) ± 1.0	4.9 (8.1) ± 3.2
**$TM_{706-757}$**	2.2 (3.9) ± 1.1	2.6 (3.9) ± 1.4	2.4 (4.3) ± 1.3

For each protein segment the number of residues is indicated. Statistics are computed on the Cα atoms for each fragment and the values relative to the segment C are calculated without the contribution of the transmembrane domain. The model after the minimization was used as a reference structure. Values are in Å: Mean (last frame) ± standard deviation.

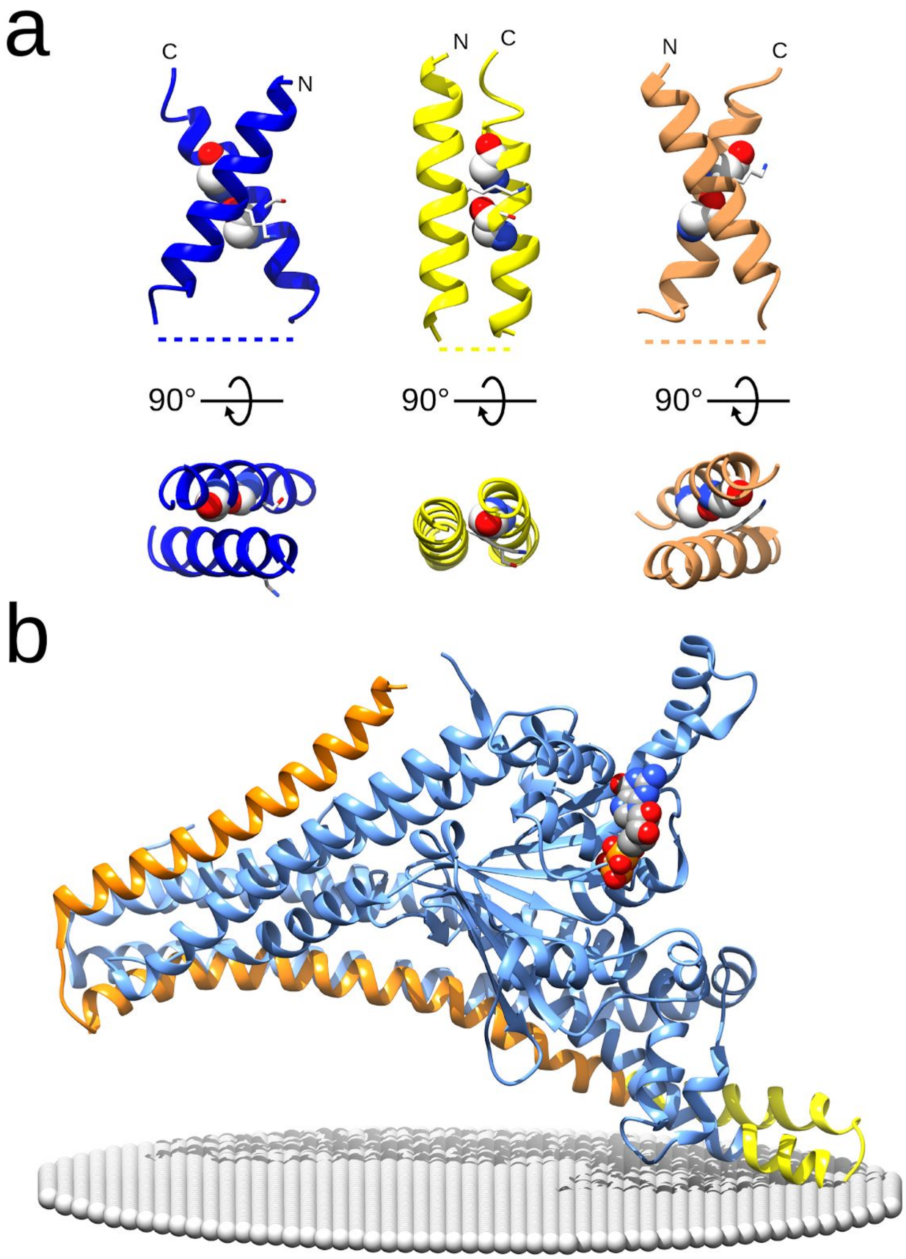

Supplementary Figure 8 | (a) *Ab initio* prediction for the TM helical dimer in Fzo1 using the PREDDIMER server. The models are ordered from the left according to their *Fscor* computed by PREDDIMER [24], 3.113, 3.100 and 2.647, respectively, with associated crossing-angle χ of 119.7°, 175.1° and -129.7°, respectively. The glycines within the motif GxxxG are in the space-filled representation and residues Lys716 and Ser746 are depicted in stick form. **(b) Prediction of the BDLP template orientation with respect to a membrane.** The crystal structure PDB-Id 2J68 [16] has been submitted to the PPM web server [67]. The structure is represented in ribbon mode, the GDP nucleotide in a space-filled representation. The N- (res 2–571) and C-terminal (res 607–695) regions exposed outside of the membrane are in *cyan* and *orange*, respectively. The paddle region (res 572–606) is depicted in *yellow*. The membrane layer is represented by dummy atoms. The predicted embedded residues are 574, 577, 581 and 583, suggesting that BDLP may be a peripheral membrane protein.

Supplementary Table 4 | Residue contacts for the TM domains in the membrane.

TM1	TM2		
	**Fzo1.I**	**Fzo1.II**	**Fzo1.III**
Leu707	Ile753 (91%)	Leu757 (73%)	Ile753 (56.3%)
Thr708	Leu750 (56.8%)	Leu757 (39.8%)	Leu750 (63.3%)
Leu709	Leu750 (70.3%)	Ile753 (62.3%)	
Phe711	Ile753 (61.4%)	Ile753 (88.2%)	Ile753 (92.9%)
Leu712	Ser746 (36%)	Leu750 (55.2%)	Leu750 (51.8%)
Thr715	Gly749 (44.5%)	Ile753 (99.8%)	Gly749 (41.3%)
Lys716	Ile742 (55.7%)	Ser746 (68.5%)	Ser746 (73.7%)
Gly719	Val741 (67.4%)		Ile742 (88%)
Asn720			Ile742 (91%)
Leu723	Val741 (85.7%)	Ile742 (93.2%)	Ile742 (72.8%)

The Table shows for each monomer in the transmembrane segment which are the corresponding partners between the two TM helices. For each residue position the number of contacts identified are summed and the persistence along each trajectory is indicated. The analysis is conducted without considering the H and the polar atoms N and O. The single common interaction between the replicas is highlighted. TM1 (res 706–726); TM2 (res 737–757).

Supplementary Table 5 | Interactions between protein and membrane.

	Fzo.I	Fzo.II	Fzo.III
POPE	Ser567		Ser567
	Arg759	Arg759	Arg759
	Lys736		
	Leu737		
	Tyr562		Tyr562
	Asp756	Asp756	Asp756
	Asp546	Asp546	
		Ser738	
		Ser732	
			Asp563
			Ile565
			Thr568
POPC	Gly559	Gly559	Gly559
	Lys736		Lys736
	Gly557		Gly557
	Leu558	Leu558	Leu558
		Lys703	
			Arg759
			Lys727

Residues interacting through H-bonds over 50% of persistence are indicated. The common interactions between the replicas are highlighted. POPE, palmitoyl-oleoyl-phosphatidylethanolamine; POPC, palmitoyl-oleoyl-phosphatidylcholine.

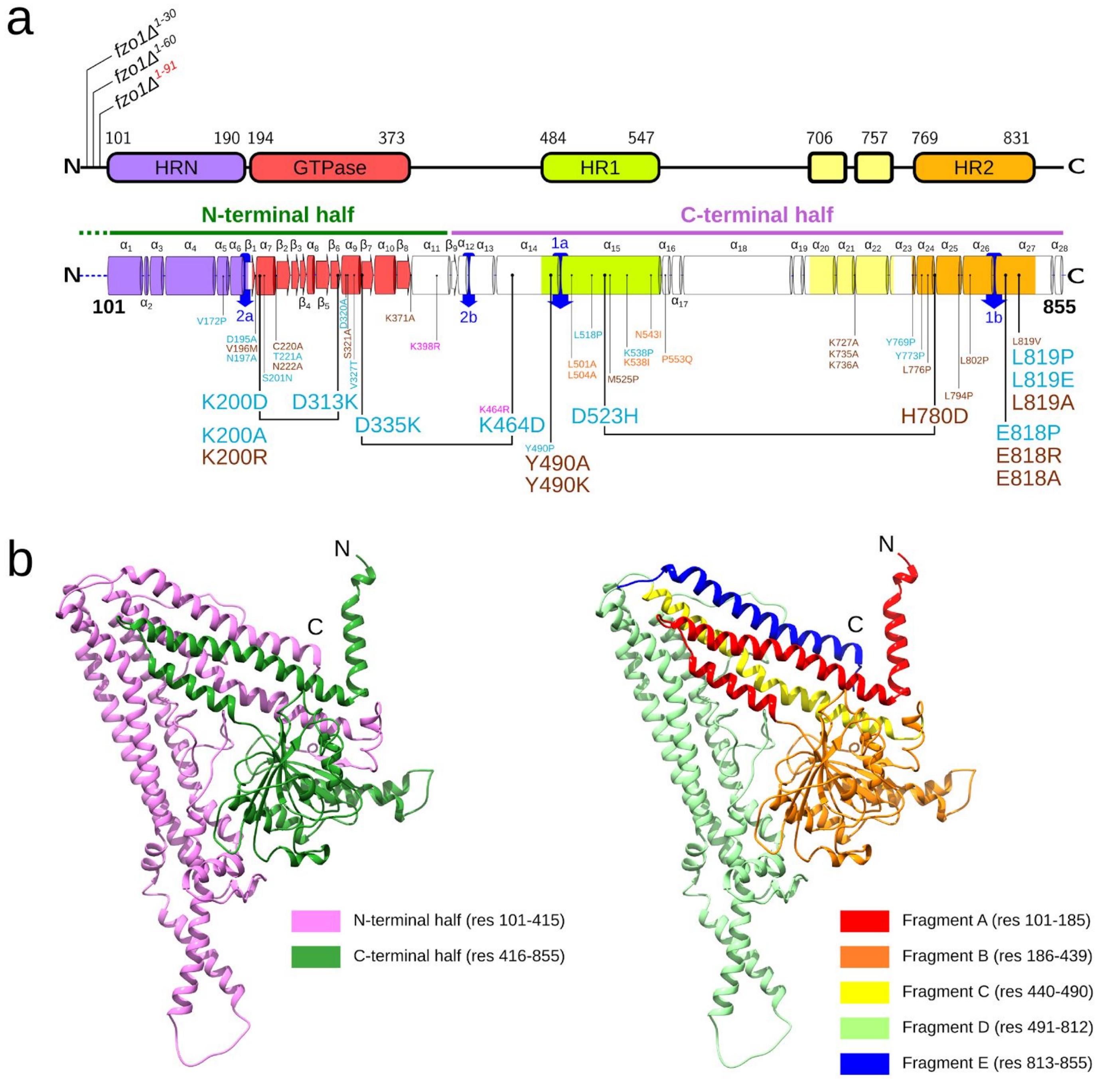

Supplementary Figure 9 | (a) Cartoon representation of the Fzo1 model and its functional domains. (top) Residue numbers delimiting the domains, (bottom) secondary structure elements are annotated with the Fzo1 mutations performed in this study. The Fig. 4 in main text has been replicated in (a) and extended with data on available mutants across Fzo1 functional domains from the literature [9, 11, 13, 15, 33, 86]. The point mutations performed in this study are highlighted by larger font size at the very bottom. The color code is *cyan*, loss of function (LOF), *maroon*, yeast phenotype is analogous to the wild-type, *orange*, point mutations that cause a severe LOF only when associated; *magenta*, residue involved in post-translational modification. Residues considered for the charge swap strategy are connected by a bar. Putative hinge regions are indicated by *blue* arrows. N- and C-terminal halves are indicated above the secondary structure plot (*green* and *pink*, respectively). The topology diagram was generated with the HERA program [83]. **(b) Level of structuration discussed in this study for the Fzo1 model.** The structures refer to the model after the equilibration phase presented also in Fig. 1d. Left, the annotation according to the N- (res 1–415, *pink*) and C-terminal (res 416–855, *green*) halves. Right, subdomains across the hinge regions considered in this study.

Supplementary Table 6 | Fzo1 mutants performed in the present study.

Category	Residue	Phenotype
Deletion	*fzo1Δ*$^{1-30}$	wild-type
	fzo1Δ$^{1-60}$	wild-type
	fzo1Δ$^{1-91}$	abolish respiration
Point mutation	*K200A*	abolish respiration
	K200D	abolish respiration
	K200R	wild-type
	D313K	abolish respiration
	D335K	abolish respiration
	K464D	abolish respiration
	Y490A	wild-type
	Y490K	wild-type
	D523H	abolish respiration
	H780D	wild-type
	E818A	wild-type
	E818P	abolish respiration
	E818R	wild-type
	E819A	wild-type
	E819P	abolish respiration
	E819E	abolish respiration
Double mutant (charge swap)	*K200D-D313K*	abolish respiration
	D335K-K464D	restore respiration
	D523H-H780D	restore respiration

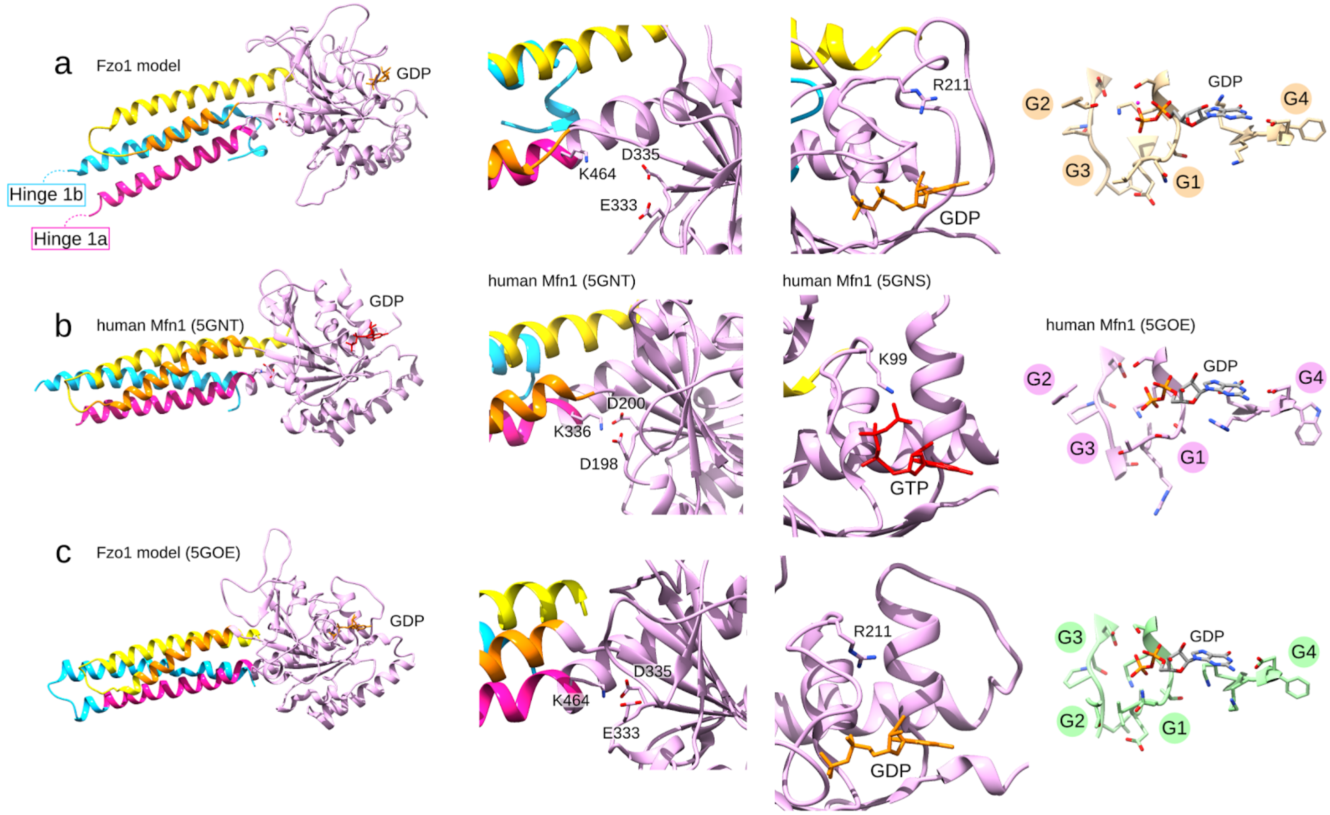

Supplementary Figure 10 | Comparison between Fzo1 model and Minimal GTPase Domain (MGD) from Mfn1. (a), (b) and (c) Fzo1 model, Mfn1 crystal fragment (PDB-Id 5GNT [18]) and the partial Fzo1 model based on human Mfn1 (PDB-Id 5GOE [19]), respectively. From left to right MGD domain, homologous salt bridges identified in Fzo1 as well as in the human Mfn1, detail of the GTPase domain showing the homologous residues that may compensate the nucleotide coordination in the G1/P-loop mutant (i.e. K200A and K88A, Fzo1 and Mfn1, respectively) and detail of the GTP binding site indicating the G1–G4 motifs involved in nucleotide stabilization, respectively. The structures were superposed with each other over the correspondent Cα for each fragment using UCSF Chimera [61]. Putative hinge 1a and 1b in Fzo1 are indicated and nucleotides are represented in *orange* and *red* stick for yeast and human, respectively. GTPase domain, *pink*; helices at N-terminal (*yellow* and *orange*) and C-terminal (*magenta*) of the GTPase domain are colored as in Qi et al., 2016 [18] for clarity.

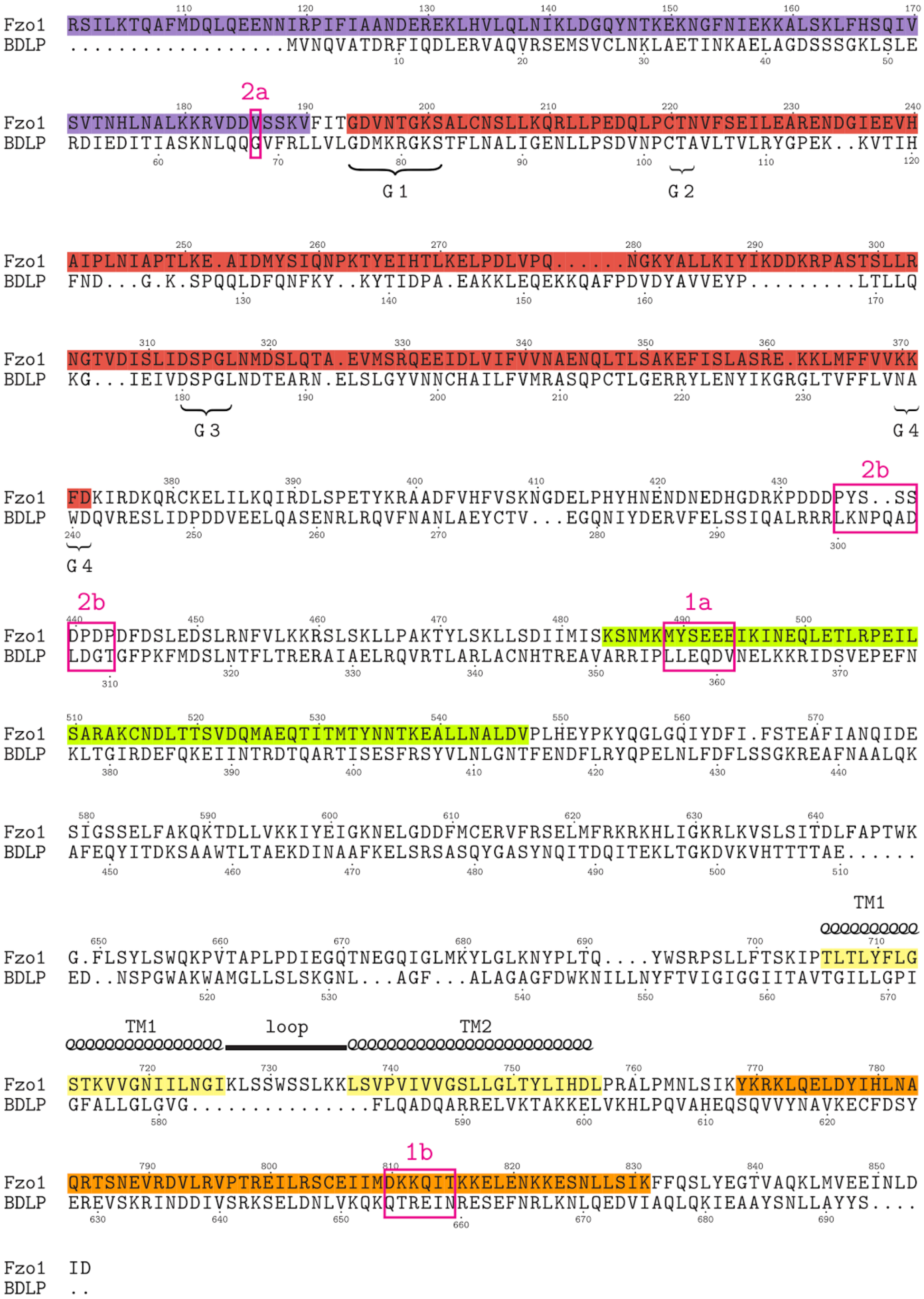

Supplementary Figure 11 | Target-template alignment after the refinement procedure. HRN (N-terminal heptad repeat) *violet*, res 91-190; GTPase domain *red*, res 194-373; HR1 (heptad repeat 1) *green*, res 484-547; transmembrane domain *yellow*, res 706-757; HR2 (heptad repeat 2) *orange*, res 769-831. Frames in *magenta* indicate putative hinges with respect to the BDLP template. In order from the N-terminal: hinge 2a (res 186) hinge 2b (res 435, 441, 443) hinge 1a (res 489-494) and hinge 1b (res 810-815).

Supplementary Table 7 | Analysis of the distances between predicted *a-d* positions in Fzo1 heptad repeats.

a,d	*a',d'*	Fzo1.I	Fzo1.II	Fzo1.III
M487	L828	12.99 ± 1.69	14.27 ± 1.35	15.43 ± 0.76
Y490	S825	14.88 ± 1.93	16.12 ± 1.68	17.72 ± 0.68
E494	N821	20.92 ± 1.92	22.64 ± 1.44	24.05 ± 1.30
I497	E818	19.82 ± 1.01	19.97 ± 1.24	21.88 ± 1.29
L501	I814	18.78 ± 1.24	17.86 ± 1.72	19.79 ± 1.27
L504	K811	21.32 ± 1.22	20.45 ± 1.84	20.30 ± 1.98
I508	I807	18.18 ± 0.57	17.94 ± 0.94	18.24 ± 0.84
A511	S804	19.74 ± 0.57	19.73 ± 0.73	19.91 ± 0.73
C515	E800	19.17 ± 0.81	19.44 ± 0.90	19.70 ± 1.31
L518	P797	20.28 ± 0.59	20.29 ± 0.57	20.25 ± 0.72
V522	V793	19.06 ± 0.68	19.41 ± 0.47	19.19 ± 0.75
M525	V790	19.73 ± 0.85	19.59 ± 0.72	18.46 ± 1.59
T529	T786	18.73 ± 1.12	18.75 ± 0.51	17.88 ± 1.14
M532	A783	18.99 ± 1.24	19.15 ± 0.66	18.30 ± 1.08
N536	I779	18.32 ± 1.73	19.15 ± 0.66	18.30 ± 1.08
E539	L776	18.06 ± 1.44	17.73 ± 0.68	17.44 ± 0.56
N543	K772	19.41 ± 1.13	19.12 ± 0.87	19.01 ± 0.87
D546	Y769	20.08 ± 1.46	18.16 ± 0.70	19.62 ± 1.43

Distances are in Å. The residue at position *a* on one chain is packed against the corresponding residue at position *d* on the other chain to form *a-d* packing. The standard deviation is indicated.

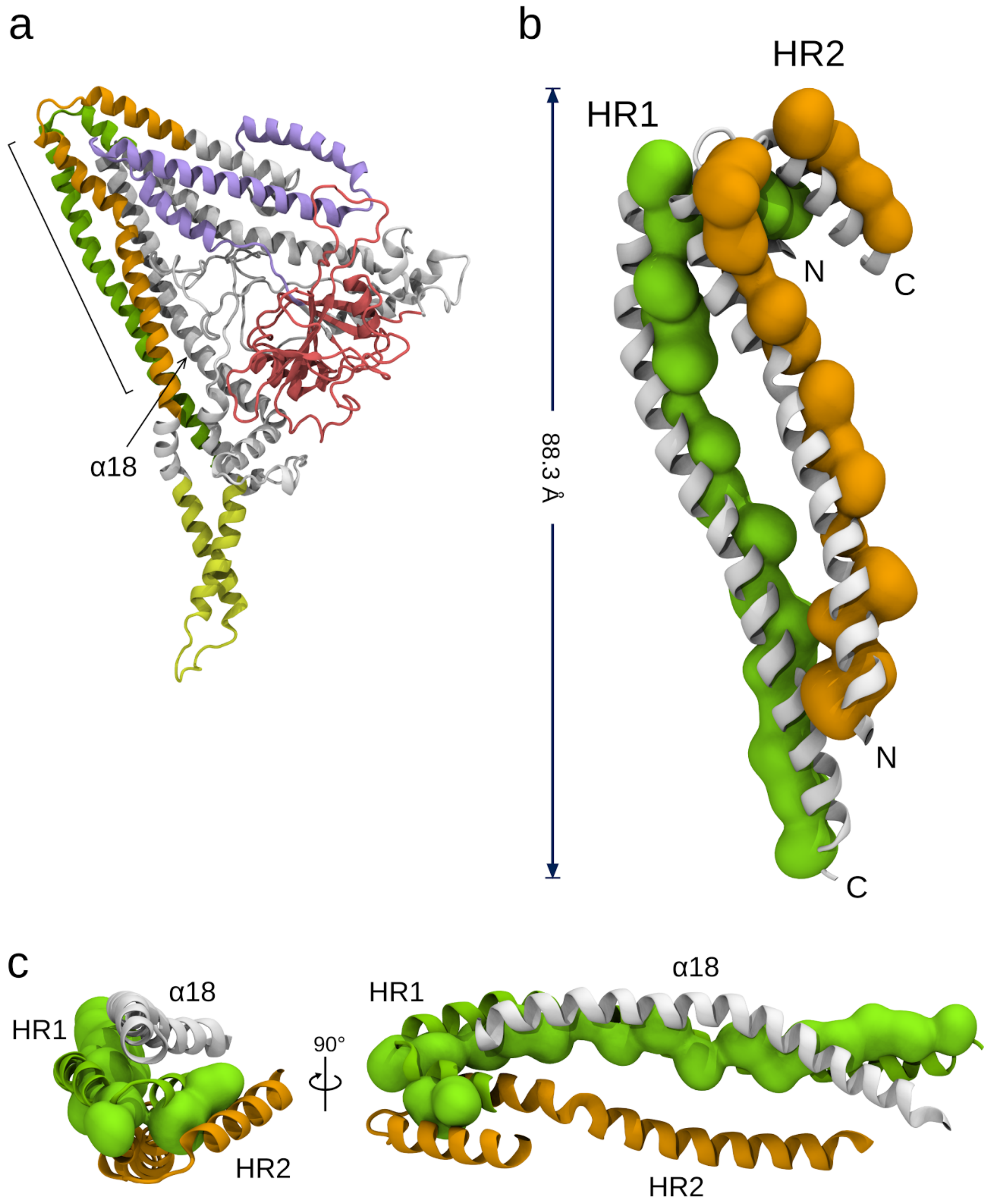

Supplementary Figure 12 | Heptad repeat domains HR1 and HR2 in the Fzo1 model. (**a**) Fzo1 model after the cluster analysis in the Fzo1.I trajectory with the α18 helix labelled. (**b**) HR1 (*green*) and HR2 (*orange*) domains as indicated in (a). The surface represents the side-chains of the predicted *a-d* positions in the heptads. (**c**) Helix bundle created by the HR1, HR2 and α18 (*white*). The latter does not exhibit heptad periodicity and in our model is in close contact with the HR1 towards its *a-d* positions (*green* surface). The PCOILS algorithm [53] has been used to predict heptad periodicity (see Methods).

Supplementary Table 8 | Detected salt bridges between the heptad repeat domains in the Fzo1 model.

HRN/HRN	HR1/HR1	HR2/HR2	HR1/HR2
Glu116 (n)-Arg120 (n+4)	Lys488 (n)-Glu492 (n+4)	Asp792 (n)-Arg795 (n+3)	Arg505-Glu806
Glu116 (n)-Arg130 (n+14)			Glu494-Lys812
Asp128 (n)-Lys132 (n+4)			
Asp143 (n)-Lys180 (n+37)			

The spacing position for the interhelical interactions is indicated. The persistence is above 60% over the trajectories, the distance cut off is up to 6.5 Å.

Supplementary Table 9 | Target-template homologous residues forming hydrogen bonds with the GDP nucleotide.

BDLP residue	Donor-acceptor distance (Å)	GDP atom	Donor-acceptor distance (Å)	Fzo1 residue
Lys79.N	3.04	O2B	2.99	Asn197.N
Arg80.N	3.38	O2B	3.43	Thr198.N
Ser83.N	2.44	O1B	2.59	Ser201.N
Thr84.N	3.14	O1A	3.15	Ala202.N
Thr84.Oγ1	3.19	O1A		
Asn238.Nδ2	3.21	O6	3.18	Lys370.Nζ
Asn238.Oδ1	3.28	N7		
Ala239.N	3.25	O6	3.26	Lys371.N
Asp241.Oδ1	2.73	N2	2.70	Asp373.Oδ2
Asp241.Oδ2	2.81	N1	2.80	Asp373.Oδ1
Ser292.N	3.04	O6	3.04	Gly427.N
Ile293.N	3.27	O6	3.21	Asp428.N

Donor-acceptor distances are calculated with LigPlot+ [52] using a distance cut-off of up to 3.5 Å.

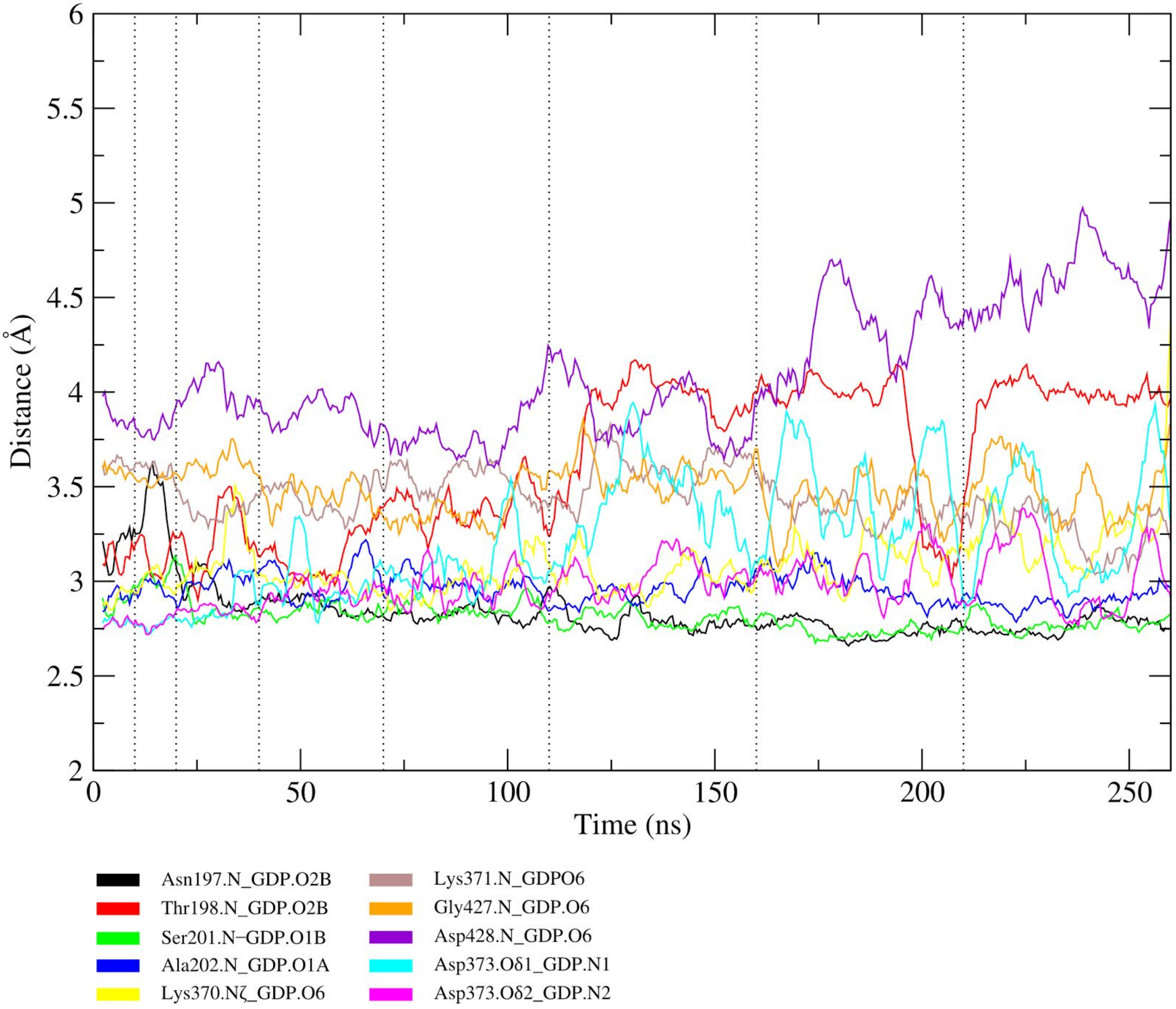

Supplementary Figure 13 | Time series of hydrogen-bond Donor-acceptor distances monitored during the equilibration phase. The colour code is indicated. The dotted lines represent the equilibration steps of varying position restrains on the Fzo1 protein backbone and side-chain atoms (see Methods).

Supplementary Table 10 | Fzo1-human mitofusin 1 homologous residues forming hydrogen bonds with the GDP nucleotide. See next page for caption.

	Fzo1 Model (this study)	5GNR [18]	5GNT [18]	5GOE [19]
**Retained**	Asn197N.O2B	Ser85N.O1B<br>Ser85N.O3B<br>Ser85Oγ.O3B<br>Ser85Oγ.O3A	Ser85Oγ.O2B<br>Ser85Oγ.O3B<br>Ser85Oγ.O1A<br>Ser85Oγ.O3A<br>Ser85Oγ.O5′	Ser85N.O2B<br>Ser85N.O3B<br>Ser85Oγ.O2B<br>Ser85Oγ.O3B<br>Ser85Oγ.O3A<br>Ser85Oγ.O5′
	Ser201N.O1B	Ser89N.O2B<br>Ser89Oγ.O2B<br>Ser89Oγ.O1A<br>Ser89Oγ.O2A<br>Ser89Oγ.O3A	Ser89N.O1B<br>Ser89Oγ.O1B<br>Ser89Oγ.O3B<br>Ser89Oγ.O1A<br>Ser89Oγ.O2A<br>Ser89Oγ.O3A	Ser89N.O1B<br>Ser89N.O3B<br>Ser89Oγ.O1B<br>Ser89Oγ.O1B<br>Ser89Oγ.O1A
	Ala202N.O1A	Ser90N.O2A<br>Ser90Oγ.O2A<br>Ser90Oγ.O3A<br>Ser90N.O2B<br>Ser90Oγ.O2B	Ser90N.O2A<br>Ser90Oγ.O2A<br>Ser90Oγ.O3A<br>Ser90Oγ.O1B<br>Ser90N.O1B	Ser90N.O2A<br>Ser90Oγ.O2A<br>Ser90Oγ.O3A<br>Ser90N.O1B<br>Ser90Oγ.O1B<br>Ser90N.O3B<br>Ser90Oγ.O5′
**Average behavior**	Thr198N.O2B	Ser86N.O2B<br>Ser86Oγ.O2B<br>Ser86Oγ.O3A	Ser86N.O2B<br>Ser86Oγ.O2B<br>Ser86Oγ.O3A	Ser86Oγ.O3B
	Lys370Nζ.O6	Asn237Nδ2.N7	Asn237Nδ2.O6<br>Asn237Nδ2.N7<br>Asn237Nδ2.O3A	Asn237Nδ2.O6
	Lys371N.O6	Arg238N.O6<br>Arg238Nε.O4′<br>Arg238Nε.O5′<br>Arg238NH2.O5′<br>Arg238NH2.O4′	Arg238N.O6<br>Arg238Nε.O4′<br>Arg238Nε.O5′<br>Arg238NH2.O5′	Arg238N.O6<br>Arg238NH1.O3′<br>Arg238NH2.O3A<br>Arg238NH2.O4′
	Asp373Oδ1.N1<br>Asp373Oδ2.N2	Asp240Oδ1.N2<br>Asp240Oδ2.N2<br>Asp240N.N1<br>Asp240N.O6	Asp240Oδ2.N2<br>Asp240N.N1<br>Asp240N.O6	Asp240Oδ2.N2<br>Asp240Oδ1.N2<br>Asp240N.N1<br>Asp240N.O6
**Not retained**	Gly427N.O6	Lys286N.O6<br>Lys286N.N7<br>Lys286Nζ.O2′<br>Lys286Nζ.O3′	Lys286N.O6<br>Lys286N.N7<br>Lys286Nζ.O3′	Lys286N.O6<br>Lys286N.N7<br>Lys286Nζ.O2′<br>Lys286Nζ.O3′
	Asp428N.O6	Glu287N.O6	Glu287N.O6	Glu287N.O6<br>Glu287N.N1

Supplementary Table 10 | Homologous network of hydrogen bond identified in the human mitofusin 1. Homologous residues were identified in the initial target-template alignment (see Methods) as well as after the superposition of the structures indicated. The Fzo1 model represents the centroid of Fzo1.I (see the main text). Residues were subdivided in the three categories discussed in the text. In *blue*, the analogous interactions, in *cyan*, interactions retained on the same GDP atom. H-bond were identified using UCSF Chimera [61] (distance cut-off of 3.5 Å and up to 30 degree off-axis angle).

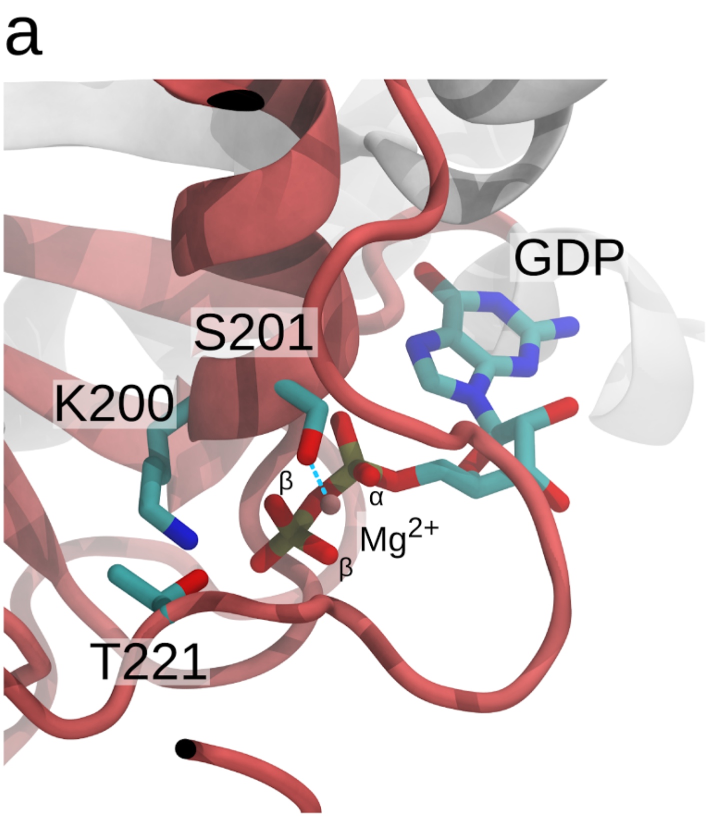

Supplementary Figure 14 | Coordination of the bound magnesium in the GDP binding site. (**a**) Fzo1 model after the minimization phase, in which the Ser201 (Ser89 in human Mfn1) directly coordinates the cation with a distance of 1.96 Å, as suggested also in the fragment crystal structure from Mfn1 [19]. Similarly, the homologous Ser41 in dynamin was proposed to act in concert with Lys44 (Lys200 in Fzo1) and Thr65 (Thr221 in Fzo1) in coordinating the bound magnesium, to stabilize the developing charge in the transition state of GTP hydrolysis [37, 87]. We thus added a magnesium ion in the nucleotide binding site of the Fzo1 model (see Methods in main text), which revealed that Ser201 of Fzo1 possibly plays a role in Mg^{2+} binding. (**b**) The structure represents the result of the cluster analysis from the trajectory Fzo1.I. During the simulation time the Ser201 oxydril group was subsequently stabilized over the average of 4.2 ± 0.19 Å between the trajectories. In particular, we observed a change in the coordination from 3 oxygens (1 from α and 2 from β phosphates, see a) to 2 oxygens (1 from α and 1 from β phosphates, see b) with the remaining coordinations being supplied by water molecules. Furthermore, analysis of minimum distances in combination with the number of contacts showed that the water molecules coordinating the Mg^{2+} at the equilibration phase, remained in place over the simulation time for each replica. Although the placement of the bound magnesium was suggested by available crystal structures (see Method), a template to correctly position the Mg^{2+} ion for the mitofusin Fzo1 is currently lacking. However, our results suggest a role for this cation in ligand accommodation within the binding pocket as recently suggested for human Mfn1 [18, 19].

Supplementary Table 11 | Fzo1 membrane localization prediction.

Protein segment	Fzo1 UniprotKB (P38297)	MEMSAT-SVM	OCTOPUS	TMpred
N-term OUT (exposed to cytosol)	1–705	1–703	1–734	1–706
TM 1	706–726	704–719		707–731
intermembrane loop (exposed to intermembrane space)	727–736	720–736		732–736
TM 2	737–757	737–755	735–756	737–755
C-term OUT (exposed to cytosol)	758–855	756–855	757–855	756–855

The different predictors used are UniprotKB [41], MEMSAT-SVM [54], OCTOPUS [55] and TMpred [56]. TM1 and TM2 are the first and the second transmembrane helix, respectively.

```
                            10                  20              30          40
S.cerevisiae   MSEGKQQFKD.SNKPH.......KDSTDQD.DDAATI....VPQT...LTYSRNEGHFLG
K.lactis       MSQKEISQNS.N.RR..........SSKYE.DELSND....APFE...ISSHQLESSTTL
E.gossypii     MSEDKRNGKD.A..............................PWE...LSYGRDGAGFNG
S.pombe        MEKSARQLSV.AEQNG.......E..........................SGRLNNGYT.
M.oryzae       MSQDSAHSSD.ATGPAPGARSNGEGS.....STATNETPSSVPSGP..PSYMTVGTGSTS
N.crassa       MSQDYYPSKGKAPQQH....EDGEPREFDDGAEHHPQ....VPGAPTTPAYMTVGTGSTS

                   50                  60         70            80
S.cerevisiae   ...SNFHGVT...........DD.RTTLFDGEEGRREDDLL....PSLRSSNSKAHLISS
K.lactis       .TDVQMS...................NLLQRH.............SSHRRDRSRDHMFYS
E.gossypii     ...DDLD...................KFLEHG.............SKAARRASNEQLVSS
S.pombe        .................................................SNNIQQYKDET
M.oryzae       EHAHRLQALLDNDSGYGGSIAGD.GAP.LIGASGDHHQA..........LVDPDRRMQAG
N.crassa       QHAARLQAMLDNDSGYGGSIAGDSRAP.SHWDSAVHHDSPLPTPTTATHDDDANRRLQAG

                  90       100       110       120
S.cerevisiae   QLSQWNYNNNRVLLKRSILKTQAFMDQLQEENNIRPIFIAA...................
K.lactis       HLTQQSYSLNRNSLLDSITVVRPLINDLITENNQRAIYIPE...................
E.gossypii     HLSQWNYNQNRGALMQGIEEASELVSDLVRENDERPMHVPD...................
S.pombe        NRHQFEYNQNRNQLLRSIHIIQNLLNELDNYVDRSDCLFHSVWRT..DKEKSKFSGN...
M.oryzae       ATHQLYYNSHRVALARSINLAIQLLKGLREMNAKWPAHYPSVQGT..DTPTSPRPGNLRH
N.crassa       AVHQLWYNQHRVTLGRSINTVVELLKKLQEMNVTWPAHYPSVQRAVLDEPNNYGPPGLHR

                                                                      130
S.cerevisiae   .......................................................NDERE
K.lactis       .........................................................HSE
E.gossypii     ...........................................................N
S.pombe        .....................................................YYPFSPS
M.oryzae       SFSSVGEFAASATAASHPRELRRSLTSVEDIGVQDAESSKAAERRQAAAEPRLVSPQISR
N.crassa       SSTMGADFP....PPPSPHSLRRSMTTGDDHA..EPESSRAAERRNTSSEPRLVSPQIAQ

                         140       150       160       170       180       190
S.cerevisiae   KLHVLQLNIKLDGQYNTKEKNGFNIEKKALSKLFHSQIVSVTNHLNALKKRVDDVSSKVF
K.lactis       SLDVLELKVKLDGREN......MQLDKSALAQLFKTQALSAIDHLINLQTRVQDTSSKVF
E.gossypii     DLQILQVSLRLDGNWKD..D..LTLDKEALAQIFKTRATSALEHLAKLLVRVQDTSSKVF
S.pombe        KMNVITIDLSLRSSSTADEKLISQLGEEAHESLLKVHIEKANKHLFSLFSRVEDTSSKIL
M.oryzae       EFSILKLDLKLGSLHQT..ELVHSLEKGSVASLLDGKIGSSIKHLQSLRERIEDTSSKVL
N.crassa       EFSVLKLDLNLGSLHQA..DLVHSLQKESVASLLDGKIRSSIKHLYSLRERIEDTSSKVL

                         200       210       220       230       240       250
S.cerevisiae   ITGDVNTGKSALCNSLLKQRLLPEDQLPCTNVFSEILEARENDGIEEVHAIPLNIAPTLK
K.lactis       ITGDLNSGKSTLCNAFLRKKVLPEDQLPCTNVFCEILEARENGNMERVHAIPKTVATNVK
E.gossypii     ITGDLNAGKSTLCNALLRKRLLPEDQLPCTNVFCEILEARENENVEQVHAIPVSIAATVK
S.pombe        ITGDLNAGKSTLCNALVHKDILPEDQQPCTEVFCEVHDAELNDGKDCVHAIPHG......
M.oryzae       VTGDLNAGKSAFCNALLRRKILPEDQQPCTSIFCEVLDARENSGLEEVHAVHKE......
N.crassa       VTGDLNAGKSTFCNALLRRKVLPEDQQPCTSIFCEVLDARENGGIEEVHAVHRD......

                         260       270       280       290       300       310
S.cerevisiae   EAIDMYSIQNPKTYEIHTLKELPDLVPQNGKYALLKIYIKDDKRPASTSLLRNGTVDISL
K.lactis       DASVLYDMRDRSTYEDYTLDKLDQLVYDNDHYILLKIYIKDDKRPVDSSLLRNGTADISL
E.gossypii     ...........EAYDAYNILDQTTIRYS.HLRSLIKIYIRDDQRPAESSLLRNGTADIAL
S.pombe        ...LTYSHTDSSTYKVFPIEDLKRLVYETENWSMLIVYVND.GRPAHESLLHNGITDIAL
M.oryzae       ...AIYDRNDESTYDVFSLSDLEKIVIDNETYLQCKVYVKD.VRTIDESLLNNGVVDIAL
N.crassa       ...AIYDRHDEATYDVYSLKELERIVTDNETYQQCKIYIRD.ARTIDESLLNNGVVDIAL

                         320       330       340       350       360       370
S.cerevisiae   IDSPGLNMDSLQTAEVMSRQEEIDLVIFVVNAENQLTLSAKEFISLASREKKLMFFVVKK
K.lactis       IDSPGLNMDSVKTTEVMSRQEEIDLVVFVVNAENQLTLSAREFITMASREKKLMFFVINK
E.gossypii     IDSPGLNMDSVQTTEVMSRQEEIDLVIFVVNAENQLTLSGKEFISTASKEKKLMFFVVNK
S.pombe        IDAPGLNTDSMKTTSVFACQEEIDVVVFVVNAENHFTLSATDFLRNASTEKSHIFIIVNK
M.oryzae       IDAPGLNSEMTKTTAVFARQEEIDVVVFVVSAANHFTISAQDFISVAAAEKAYLFIVVNQ
N.crassa       IDAPGLNMDTTKTTAIFARQEEIDVVVFVVSATNHFTQTATEFIRAAAAEKAYLFIVVNG

                         380       390       400       410       420
S.cerevisiae   FDKIRDKQRCKELILKQIRDLSPETYKRAADFVHFVSKNGDELPHYHNEN.........D
K.lactis       FDHIKDKDRCKKLILDQIKEISPETYKQNSEFVHFVTSHGKIPHEGENRP..........
E.gossypii     FDHIKDKQRCKKLILDQIKEISPETYKQSTEFVHFISSEGVFIDDSNGNP..........
S.pombe        FDNIRDKERCKRLILEQIHTLSPGTFADAKDLVHFVSCRVAR..................
M.oryzae       FDNIRDKERCQKMILDRVKMMSPRTYKEASELVHFVSSNAVPVAPPPPPPGGSGGSGSSS
N.crassa       FDTIRDKERCQKLILNQVRGLSPATHKEADELVHFVSSTAIPMAPSPPGGSYGGGSGSAS
```

Supplementary Figure 15 | Multiple sequence alignment of Fzo1 homologous identified in this study that belong to the FZO1 subfamily. Continue next page.

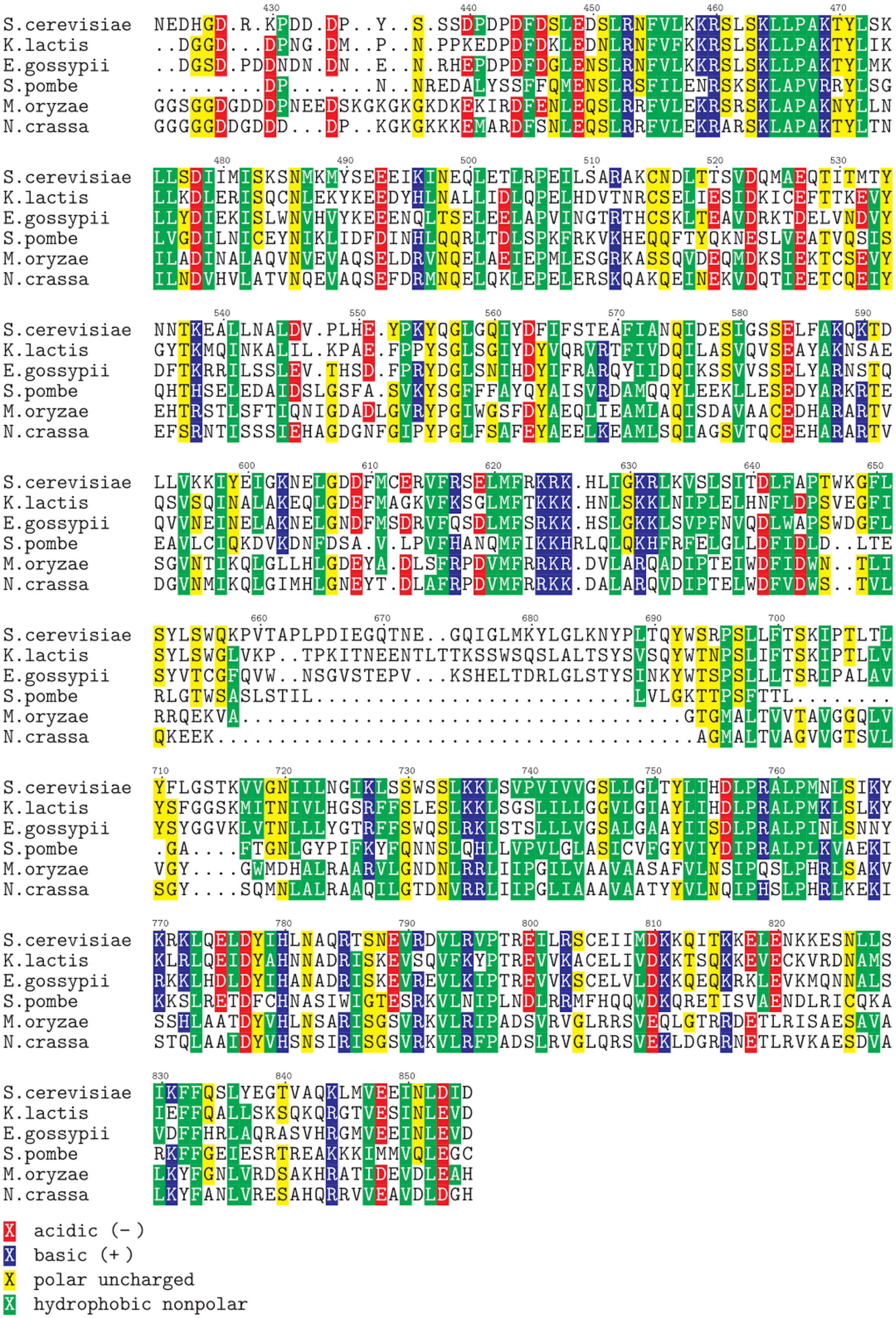

Supplementary Figure 15 | Multiple sequence alignment of Fzo1 homologous identified in this study that belong to the FZO1 subfamily. The species considered are *Saccharomyces cerevisiae*, *Kluyveromyces lactis*, *Eremothecium gossypii*, *Schizosaccharomyces pombe*, *Magnaporthe oryzae* and *Neurospora crassa*. (see also Supplementary Table 1).

Supplementary Table 12 | Characteristics of the simulated system.

Box size in $Å^3$	N water	N ions ($K^+/Cl^-/Mg^{2+}$)	N lipids (POPE/POPC)	N atoms
102,58 × 102,58 × 164,80	36919	121/120/1	156/156	163769

Values are the same for all three molecular dynamics simulations Fzo1.I, Fzo1.II and Fzo1.III.

Supplementary Table 13 | Plasmids used in this study.

Name (Collection number)	Description	Reference
pRS314	CEN, *TRP1*, Amp	84
pRS414-FZO1-Myc-FL (MC210)	CEN, *FZO1* promoter-*FZO1-9MYC*, *TRP1*, Amp	11
pRS414-1-30 FZO1-Myc-FL (MC380)	CEN, *FZO1* promoter-1-30 *FZO1-9MYC*, *TRP1*, Amp	This study
pRS414-1-60 FZO1-Myc-FL (MC381)	CEN, *FZO1* promoter-1-60 *FZO1-9MYC*, *TRP1*, Amp	This study
pRS414-1-91 FZO1-Myc-FL (MC382)	CEN, *FZO1* promoter-1-91 *FZO1-9MYC*, *TRP1*, Amp	This study
pRS314-FZO1 (MC250)	CEN, *FZO1* promoter-*FZO1*, *TRP1*, Amp	13
pRS314-FZO1 D335K (MC377)	CEN, *FZO1* promoter-*fzo1 D335K*, *TRP1*, Amp	This study
pRS314-FZO1 K464D (MC378)	CEN, *FZO1* promoter-*fzo1 K464D*, *TRP1*, Amp	This study
pRS314-FZO1 D335K-K464D (MC379)	CEN, *FZO1* promoter-*fzo1 D335K-K464D*, *TRP1*, Amp	This study
pRS414-FZO1-13Myc (MC333)	CEN, *FZO1* promoter- *FZO1-13MYC, TRP1*, Amp	31
pRS314-FZO1 K464D-13Myc (MC442)	CEN, *FZO1* promoter-*fzo1 K464D-13MYC*, *TRP1*, Amp	This study
pRS314-FZO1 D335K-K464D-13Myc (MC444)	CEN, *FZO1* promoter-*fzo1 D335K-K464D-13MYC*, *TRP1*, Amp	This study
pRS314-FZO1 K398R-13Myc (MC334)	CEN, *FZO1* promoter-*fzo1 K398R-13MYC*, *TRP1*, Amp	This study
pRS314-FZO1 D523H (MC400)	CEN, *FZO1* promoter-*fzo1 D523H*, *TRP1*, Amp	This study
pRS314-FZO1 H780D (MC396)	CEN, *FZO1* promoter-*fzo1 H780D*, *TRP1*, Amp	This study
pRS314-FZO1 D523H-H780D (MC397)	CEN, *FZO1* promoter-*fzo1 D523H-H780D*, *TRP1*, Amp	This study
pRS314-FZO1 L819A (MC434)	CEN, *FZO1* promoter-*fzo1 L819A*, *TRP1*, Amp	This study
pRS314-FZO1 L819E (MC433)	CEN, *FZO1* promoter-*fzo1 L819E*, *TRP1*, Amp	This study
pRS314-FZO1 L819P (MC411)	CEN, *FZO1* promoter-*fzo1 L819P*, *TRP1*, Amp	13
pRS314-FZO1 E818P (MC406)	CEN, *FZO1* promoter-*fzo1 E818P*, *TRP1*, Amp	This study
pRS314-FZO1 E818R (MC407)	CEN, *FZO1* promoter-*fzo1 E818R*, *TRP1*, Amp	This study
pRS314-FZO1 E818A (MC408)	CEN, *FZO1* promoter-*fzo1 E818A*, *TRP1*, Amp	This study
pRS314-FZO1 Y490A (MC431)	CEN, *FZO1* promoter-*fzo1 Y490A*, *TRP1*, Amp	This study
pRS314-FZO1 Y490K (MC432)	CEN, *FZO1* promoter-*fzo1 Y490K*, *TRP1*, Amp	This study
pRS314-FZO1 Y490K-L819E (MC432)	CEN, *FZO1* promoter-*fzo1 Y490K-L819E*, *TRP1*, Amp	This study
pRS314-FZO1 D313K (MC390)	CEN, *FZO1* promoter-*fzo1 D313K*, *TRP1*, Amp	This study
pRS314-FZO1 K200D (MC391)	CEN, *FZO1* promoter-*fzo1 K200D*, *TRP1*, Amp	This study
pRS314-FZO1 D313K-K200D (MC392)	CEN, *FZO1* promoter-*fzo1 D313K-K200D*, *TRP1*, Amp	This study
pRS314-FZO1 K200A (MC445)	CEN, *FZO1* promoter-*fzo1 K200A*, *TRP1*, Amp	13
pRS314-FZO1 K200R (MC427)	CEN, *FZO1* promoter-*fzo1 K200R*, *TRP1*, Amp	This study

Supplementary Table 14 | *Saccharomyces cerevisiae* strains used in this study.

Name	Genotype	Reference
FZO1 (MCY571)	*MATα ura3-1 trp1-1 leu2-3,112 his3-11,15 can1-100 fzo1Δ::LEU2* pRS416-FZO1	This study
FZO1 mdm30 (MCY585)	*MATα ura3-1 trp1-1 leu2-3,112 his3-11,15 can1-100 fzo1Δ::LEU2 mdm30Δ::KanMX6* pRS416-FZO1	This study

Supplementary References

84. Sikorski, R. S. & Hieter, P. A system of shuttle vectors and yeast host strains designed for efficient manipulation of DNA in Saccharomyces cerevisiae. *Genetics* **122,** 19–27 (1989).

85. Waterhouse, A. M., Procter, J. B., Martin, D. M. A., Clamp, M. & Barton, G. J. Jalview Version 2—a multiple sequence alignment editor and analysis workbench. *Bioinformatics* **25,** 1189–1191 (2009).

86. Amiott, E. A., Cohen, M. M., Saint-Georges, Y., Weissman, A. M. & Shaw, J. M. A mutation associated with CMT2A neuropathy causes defects in Fzo1 GTP hydrolysis, ubiquitylation, and protein turnover. *Mol. Biol. Cell* **20,** 5026–5035 (2009).

87. Saraste, M., Sibbald, P. R. & Wittinghofer, A. The P-loop--a common motif in ATP- and GTP-binding proteins. *Trends Biochem. Sci.* **15,** 430–434 (1990).
